# Five‐Year Disease Progression in Synuclein Seeding Positive Sporadic Parkinson's Disease

**DOI:** 10.1002/acn3.70323

**Published:** 2026-03-10

**Authors:** Paulina Gonzalez‐Latapi, Caroline Gochanour, Seung Ho Choi, Hyunkeun Cho, Chelsea Caspell‐Garcia, Christopher Coffey, Michael Brumm, David‐Erick Lafontant, Yuge Xiao, Thomas Tropea, John Seibyl, Caroline Tanner, Charles S. Venuto, Karl Kieburtz, Lana M. Chahine, Kathleen L. Poston, Andrew Siderowf, Kenneth Marek, Tanya Simuni, The Parkinson's Progression Markers Initiative

**Affiliations:** ^1^ The Ken and Ruth Davee Department of Neurology Chicago Illinois USA; ^2^ Department of Biostatistics University of Iowa Iowa City Iowa USA; ^3^ Division of Biostatistics University of California San Diego La Jolla California USA; ^4^ The Michael J. Fox Foundation for Parkinson's Research New York New York USA; ^5^ Institute for Neurodegenerative Disorders New Haven Connecticut USA; ^6^ Department of Neurology Yale University New Haven Connecticut USA; ^7^ Department of Neurology University of California San Francisco California USA; ^8^ Department of Neurology University of Rochester Rochester New York USA; ^9^ Department of Neurology University of Pittsburgh Pittsburgh Pennsylvania USA; ^10^ Department of Neurology Stanford University Palo Alto California USA; ^11^ Department of Neurology University of Pennsylvania Philadelphia Pennsylvania USA

## Abstract

**Objective:**

To provide a comprehensive description of disease progression in synuclein seeding assay (SAA) positive sporadic Parkinson Disease participants, using Neuronal Synuclein Disease integrated biological and functional impairment staging framework.

**Methods:**

We analyzed 5‐year longitudinal data from 345 participants recruited in the Parkinson's Progression Markers Initiative with the diagnosis of early (less than 2 years of clinical diagnosis at baseline and untreated) sporadic Parkinson's Disease, who were synuclein seeding assay positive. We assessed 5‐year progression in a spectrum of clinical and biomarker measures. We used Cox proportional hazards models to assess the association between baseline stage and time to survival, postural instability, cognitive impairment, and other meaningful milestones. Biomarker analysis included dopamine transporter binding measures, CSF‐SAA, amyloid‐beta, phosphorylated tau and total tau, as well as serum urate, and neurofilament light chain.

**Results:**

At baseline there was clear separation of participants by Neuronal Synuclein Disease Stages (23% Stage 2b, 67% Stage 3, 10% Stage 4). Participants in stage 4 at baseline had a significantly higher rate of reaching disability, postural instability, cognitive decline, and the autonomic dysfunction milestones. There was a stage‐dependent increase in dopamine deficit at baseline. There was no difference in fluid biomarkers between the stages at baseline and longitudinally.

**Interpretation:**

This study highlights the heterogeneity in the early Parkinson's Disease population defined by clinical diagnostic criteria and underscores the importance of shifting from clinical to biologically and functional impairment defined inclusion criteria for clinical trials. Biological drivers of stage heterogeneity must be further explored.

## 1 Introduction

Parkinson's Disease (PD) has been historically defined by clinical features, with a definite diagnosis only possible through the identification of alpha‐synuclein (*α*Syn) in Lewy bodies and nigrostriatal degeneration at brain autopsy [1, 2, 3]. Current diagnostic criteria are based solely on the presence of motor features [3], and diagnosis is made only once the disease process has been ongoing for more than a decade [4, 5].

Recent advances in biomarkers of αSyn pathology have revolutionized how we think about PD. The detection of neuronal αSyn through seed amplification assay in CSF (CSF‐SAA) has emerged as an in vivo biomarker of αSyn pathology, validated in multiple cohorts [6, 7, 8, 9] and against postmortem tissue [10, 11]. Immunohistochemical detection of cutaneous phosphorylated αSyn has also been proposed as a sensitive and specific clinical test for the diagnosis of synucleinopathies [12]. Based on these breakthroughs, two groups have proposed research criteria for a biological definition, classification and staging of PD. The SynNeurGe criteria classify the disease by the presence of biomarkers of pathological αSyn, neuroimaging features of neurodegeneration and the presence of PD specific pathogenic gene variants [13]. The Neuronal Synuclein Disease (NSD) criteria define the disease by the presence of pathologic αSyn as measured by a validated biomarker and participants may or may not have evidence of dopaminergic dysfunction, as measured by a validated biomarker such as dopamine transporter (DAT binding) deficit. Simuni et al. [14], have also proposed an integrated staging system for NSD (NSD‐ISS), which builds on similar efforts in other neurodegenerative diseases [15, 16]. The NSD‐ISS includes 7 stages, defined by each biomarker, presence of clinical features and their functional consequences [14]. Aligned with other neurodegenerative diseases, PD therapeutic development is transitioning to biomarkers defined enrollment and enrichment criteria to increase chances of success of biologically targeted therapeutics [17, 18].

Thus, a description of the longitudinal changes in an observational biologically defined contemporary cohort, with clinical and biological data, may inform an evolving perspective on the course of PD under contemporary management paradigms. The Parkinson's Progression Markers Initiative (PPMI) provides a uniquely comprehensive set of clinical, imaging, and biosample data from participants recruited as clinically defined de novo PD, at‐risk individuals, and healthy controls [19]. In a previous report, Simuni et al. [20] described the five‐year trajectory of the Movement Disorders Society Unified Parkinson's Disease Rating Scale (MDS‐UPDRS) and a significant, albeit modest, correlation with DAT binding over that period in a clinically defined, de novo PD cohort. However, analyzing a patient population based solely on clinical criteria may introduce heterogeneity due to underlying differences in disease biology, potentially including individuals with non‐synucleinopathies or atypical neurodegenerative processes.

The aims of our study were to: (i) describe 5‐year outcomes in CSF‐SAA + population originally enrolled in the sporadic PD (sPD) cohort in PPMI, and (ii) analyze the association between baseline NSD stage and both survival and the time to reach clinically meaningful disease milestones.

## 2 Methods

### 2.1 PPMI

PPMI is an ongoing international, multicenter, prospective cohort study initiated in June 2010 as described previously [19, 21]. The study was approved by the institutional review board at each site, and all participants provided written informed consent. The primary aim of PPMI is to identify genomic, biochemical, or imaging biomarkers of clinical progression. The detailed study protocol, manuals, biofluid collection, and storage processes are available at www.ppmi‐info.org/study‐design.

### 2.2 Study Population

Participants included in the study were those enrolled in the sPD cohort in PPMI with the following enrollment criteria: (i) presence of two or more of the following: bradykinesia, rigidity, and resting tremor OR presence of either an asymmetric resting tremor or asymmetric bradykinesia (ii) disease duration from diagnosis of ≤ 2 years, (iii) DAT binding deficit based on visual interpretation. Participants could not be treated with dopaminergic therapy or expected to need treatment within 6 months of enrollment. From this group, we selected individuals that fulfilled NSD criteria at time of or within 12 months of enrollment into PPMI, and who were recruited prior to 2020 to allow for at least 5 years of longitudinal data.

### 2.3 NSD‐ISS Staging

NSD is defined by pathologic neuronal αSyn (S) and eventual dopaminergic neuronal dysfunction (D), independent of clinical features. The NSD‐ISS integrates these biological anchors and the degree of functional impairment as follows: Stage 0 (presence of fully penetrant pathogenic variant in *SNCA* gene); Stage 1A (S+, D‐, no signs/symptoms); Stage 1B (S+, D+, no signs/symptoms); Stage 2A (S+, D‐, subtle signs/symptoms, no functional impairment); Stage 2B (S+, D+, subtle signs/symptoms, no functional impairment); Stages 3–6 (S+, D+, and clinical signs/symptoms with progressively increasing severity of functional impairment) [14] Table S1. If participants completed a follow‐up visit but were missing one or more of the components used to determine stage, the data needed to assign a stage was carried forward from the previous visit.

### 2.4 Clinical Assessments

PPMI includes a wide array of investigator completed and participant reported measures of motor, non‐motor and cognitive function. For this analysis we utilized demographic data (age, sex, race, time since diagnosis, education level), Movement Disorder Society‐Unified Parkinson's Disease Rating Scale (MDS‐UPDRS)‐Parts I‐IV [22], Hoehn & Yahr (H&Y) [23], Schwab‐England activities of daily living score (S&E) [24], age/sex adjusted University of Pennsylvania Smell Identification Test (UPSIT) score [25], Scales for Outcomes in Parkinson's disease‐ Autonomic (SCOPA‐AUT) total score [26], Geriatric Depression Scale (GDS) score [27], REM sleep behavior disorder (RBD) screening questionnaire (RBDSQ) score [28], Levodopa Equivalent Daily Dose (LEDD) [29]. Cognition was measured with the Montreal Cognitive Assessment (MoCA) [30] scores and site investigator's clinical diagnosis of cognitive state (normal cognition, mild cognitive impairment [MCI] or PD dementia [PDD]), which was not fully implemented until study year 3 [31]. The site investigator is provided a guidance document on how to assess for subjective cognitive change compared with pre‐PD state, impairment in cognitive abilities, and functional impairment due to cognitive deficits [32]. All assessments were conducted annually.

### 2.5 Imaging Biomarkers

Degree of dopaminergic dysfunction was assessed with DAT binding and quantified in two ways: as the specific binding ratio (SBR) obtained from the lowest putamen alone (operationally defined as the lower of the two putamenal sides) and as the average of SBRs obtained from the caudate and putamen (striatum) in both hemispheres. DAT binding was assessed at baseline and at years 1, 2, and 4 of the study. Imaging acquisition and analysis protocols can be found at ppmi‐info.org.

### 2.6 Biofluid Biomarkers

αSyn‐SAA was run on CSF samples. Cerebrospinal fluid was tested in triplicate for αSyn‐SAA at Amprion as previously described [33]. Briefly, samples where all 3 replicates have a maximum fluorescence (Fmax) ≥ 3000 relative fluorescence units (RFU), or exactly 2 replicates have a Fmax ≥ 3000 RFU and < 45,000 RFU, are defined “Positive” and αSyn seeds are detected, while samples where exactly 0 or 1 replicate has a Fmax ≥ 3000 RFU are called “Negative” and seeds are not detected.

CSF amyloid‐beta 1–42 (Aβ 1–42), total‐tau, and tau phosphorylated at threonine 181 position (p‐tau) were measured at baseline and annually until year 5. If measures were missing due to exceeding the upper or lower limits of detection, the limit of detection threshold was imputed. Serum neurofilament light chain (NfL) was measured at baseline and then at years 1, 2, 3 and 5. Serum urate was measured at baseline and then annually until year 5. Cutoffs for CSF A*β* 1–42 ≤ 683 pg/mL, p‐tau ≥ 13 pg/mL, and serum NfL ≥ 19.05 pg/mL were also used as variables for the analysis [34]. Biomarkers collection and analysis protocols can be found at ppmi‐info.org.

### 2.7 Survival and Progression Milestones Endpoints

The following endpoints were used to capture disease progression in our analyses: Death, postural instability (defined by H&Y ≥ 3 either on or off medication), disability (defined as S&E < 80%) and cognitive decline (defined as site investigator's clinical diagnosis of MCI or PDD). We analyzed time to reach five of the progression milestone domains as described by Brumm et al. [35], including “walking and balance”; “motor complications”; “cognition”; “autonomic dysfunction”, and “activities of daily living” (Table S2).

### 2.8 Statistical Analysis

Baseline demographic, clinical, and biological characteristics were reported for all participants and separately by baseline NSD stage. Frequency and percent were reported for categorical measures and median and interquartile range for continuous measures. To evaluate differences in baseline characteristics by NSD stage, Chi‐Square and Fisher's exact test (when appropriate) were presented for categorical measures and Kruskal‐Wallis tests for continuous variables. Nonparametric tests were calculated for continuous measures due to the small number of participants in the Stage 4 group. Longitudinal measures were reported for each annual study visit up to year 5 overall and by baseline stage. Given the significance of categorical serum NfL across stages at baseline, we modeled serum NfL using a linear mixed model with baseline stage, time from enrollment, and their interaction, and adjusted for baseline NfL, age, and sex for years 1, 2, 3, and 5. We used an unstructured working correlation structure and identity link. A Wald test was used to assess the statistical significance of the interaction term between baseline stage and time and the time effect estimate was presented with its 95% confidence limits.

To evaluate participant progression over time by baseline stage, we calculated time from enrollment in PPMI to reaching the nine endpoints of interest: death, disability, reaching H&Y stage 3 or greater, cognitive decline, walking and balance (domain 1), motor complications (domain 2), cognition (domain 3), autonomic dysfunction (domain 4), and activities of daily living (domain 6). Time to domains was calculated over the first 5 years of study participation, from enrollment to the 5‐year annual visit, or last annual visit attended prior to 5 years. Cox proportional hazards models were used to model time from enrollment to each outcome, stratified by baseline stage, and adjusting for baseline age, sex, and education (< 12 years or ≥ 12 years). If participants did not have an event during the follow‐up period, they were censored at the time of their last assessment. If participants reached any of the nine endpoints at the baseline visit, they were removed from all models. Age, sex, and education adjusted hazards ratios for comparing stage 2b to 3 and 2b to 4 are presented with their 95% Wald confidence intervals. Age, sex, and education adjusted survival curves are presented. We use a Bonferroni adjusted α‐level of 0.0056 to adjust for multiple comparisons in survival analyses.

All analyses were performed using SAS 9.4 (SAS/STAT 15.3; SAS Institute Inc., Cary, NC).

## 3 Results

### 3.1 Baseline Characteristics

Of all PD participants enrolled in PPMI prior to 2020, *n* = 345 were S+, D+ sPD, and included in the analysis. These participants also qualified for SynNeurGe criteria Figure S1. Baseline demographic and disease characteristics of the cohort as a whole and by NSD Stage are summarized in Table 1. 23% (*n* = 79) were NSD Stage 2b, 67% (*n* = 231) NSD Stage 3, and 10% (*n* = 35) NSD Stage 4. Baseline demographic characteristics were similar across the stages aside from slightly longer disease duration at enrollment in Stage 4. There was a significant stage‐dependent difference across most clinical measures, including those that were not used as stage anchors (Table 1).

**TABLE 1:** Baseline clinical and demographic characteristics.

	NSD stage at baseline				
	SAA + (*N* = 345)	Stage 2b (*N* = 79)	Stage 3 (*N* = 231)	Stage 4 (*N* = 35)	*p* (*)
Age (years), Median (IQR)	62.4 (55.2, 69.0)	61.1 (54.9, 67.2)	62.8 (55.7, 69.3)	63.4 (49.8, 69.7)	0.412
Sex, Female, *n* (%)	119 (34%)	28 (35%)	76 (33%)	15 (43%)	0.503
Education Category, < 12 Years, *n* (%)	21 (6%)	5 (6%)	15 (6%)	1 (3%)	0.880
Race, White, *n* (%)	318 (92%)	73 (92%)	211 (92%)	34 (97%)	0.530
Hispanic or Latino Ethnicity, *n* (%)	7 (2%)	1 (1%)	6 (3%)	0	0.851
Time from Diagnosis to Baseline (years), Median (IQR)	0.3 (0.2, 0.7)	0.4 (0.2, 0.6)	0.3 (0.2, 0.6)	0.6 (0.3, 1.1)	0.008
Duration of Follow Up from BL (years), Median (IQR)	5.0 (4.9, 5.0)	5.0 (5.0, 5.0)	5.0 (4.9, 5.0)	5.0 (4.0, 5.0)	0.149
UPSIT Percentile, Median (IQR)	5.5 (3.0, 13.5)	7.0 (3.0, 13.5)	5.5 (3.0, 12.5)	5.0 (2.0, 12.5)	0.632
UPSIT Percentile ≤ 15, *n* (%)	275 (80%)	66 (84%)	182 (79%)	27 (77%)	0.612
MDS‐UPDRS Part I, Median (IQR)	5.0 (3.0, 7.0)	2.0 (1.0, 5.0)	5.0 (3.0, 7.0)	13.0 (8.0, 16.0)	< 0.001
MDS‐UPDRS Part II, Median (IQR)	5.0 (3.0, 8.0)	2.0 (1.0, 2.0)	6.0 (4.0, 8.0)	14.0 (12.0, 17.0)	< 0.001
MDS‐UPDRS Part III (OFF), Median (IQR)	20.0 (15.0, 26.0)	14.0 (10.0, 20.0)	21.0 (16.0, 26.0)	26.0 (18.0, 36.0)	< 0.001
MDS‐UPDRS Total Score (OFF), Median (IQR)	31.0 (23.0, 40.0)	19.0 (14.0, 25.0)	32.0 (26.0, 39.0)	53.0 (42.0, 62.0)	< 0.001
Hoehn and Yahr, *n* (%)					< 0.001
1	153 (44%)	55 (70%)	90 (39%)	8 (23%)	
2	192 (56%)	24 (30%)	141 (61%)	27 (77%)	
On Antidepressant Medication, *n* (%)	59 (17%)	5 (6%)	40 (17%)	14 (40%)	< 0.001
On Antipsychotic Medication, *n* (%)	0	0	0	0	—
On Cognitive Enhancer Medication, *n* (%)	1 (< 1%)	0	1 (< 1%)	0	1.000
MOCA Total Score, Median (IQR)	28.0 (26.0, 29.0)	28.0 (27.0, 29.0)	27.0 (26.0, 29.0)	28.0 (26.0, 29.0)	0.042
Cognitive Categorization**, *n* (%)					1.000
Normal Cognition	71 (91%)	17 (94%)	46 (90%)	8 (89%)	
Mild Cognitive Impairment (MCI)	7 (9%)	1 (6%)	5 (10%)	1 (11%)	
RBD Screening Questionnaire Score, Median (IQR)	3.0 (2.0, 6.0)	3.0 (1.0, 5.0)	4.0 (2.0, 5.0)	7.0 (3.0, 8.0)	< 0.001
RBD Screening Questionnaire > 5, *n* (%)	86 (25%)	9 (12%)	56 (24%)	21 (60%)	< 0.001
GDS, Median (IQR)	2.0 (1.0, 3.0)	1.0 (0.0, 2.0)	2.0 (1.0, 3.0)	3.0 (2.0, 8.0)	< 0.001
SCOPA‐AUT, Median (IQR)	8.0 (5.0, 12.0)	6.0 (2.0, 9.0)	9.0 (6.0, 12.0)	14.0 (11.0, 20.0)	< 0.001
Modified Schwab & England, Median (IQR)	90.0 (90.0, 100.0)	100.0 (95.0, 100.0)	90.0 (90.0, 100.0)	90.0 (80.0, 90.0)	< 0.001

*Note: (Missing)* Race *n =* 1; Cognitive Categorization *n =* 267 (consistent data collection on this variable began after 2020); RBD Screening Questionnaire *n =* 3; SCOPA‐AUT *n =* 5. (*) Comparisons across NSD stage at baseline used Chi‐Square or Fisher's Exact tests for categorical variables and Kruskal‐Wallis tests for continuous variables.

** No participants were categorized as Dementia at baseline.

Baseline biofluid and imaging characteristics of the sPD cohort and by NSD Stage are presented in Table 2. As a group, S+ sPD participants had a median [IQR] age/sex‐expected lowest putamen SBR of 0.31 [0.26, 0.38] and a median striatum binding of 1.36 [1.17, 1.62]. Participants in NSD Stage 4 had lower striatum binding compared to stages 2b and 3 (medians of 1.30 vs. 1.52 and 1.33, respectively). Age/sex putamenal SBR was significantly different across groups, with lower (worse) scores in the NSD Stage 4 group compared to stages 2b and 3 (medians of 0.28 vs. 0.35 and 0.30, respectively). A greater percentage of participants in the NSD Stage 4 group presented a serum NfL level > 19.05 pg/mL (Table 2). There were no other differences in fluid biomarkers between NSD Stages at baseline.

**TABLE 2:** Baseline imaging and biofluid characteristics.

	NSD stage at baseline				
	SAA + (*N* = 345)	Stage 2b (*N* = 79)	Stage 3 (*N* = 231)	Stage 4 (*N* = 35)	*p* (*)
*DAT binding Measures*					
Age/Sex‐Expected Lowest Putamen SBR, Median (IQR)	0.31 (0.26, 0.38)	0.35 (0.28, 0.44)	0.30 (0.26, 0.37)	0.28 (0.21, 0.37)	0.003
Mean Striatum Binding, Median (IQR)	1.36 (1.17, 1.62)	1.52 (1.35, 1.74)	1.33 (1.15, 1.58)	1.30 (1.00, 1.62)	< 0.001
*Fluid Biomarkers*					
Serum NfL pg/mL, Median (IQR)	11.4 (8.3, 15.8)	11.5 (8.3, 15.1)	11.3 (8.4, 15.6)	13.5 (6.7, 19.5)	0.635
Serum NfL ≥ 19.05 pg/mL (N+), *n* (%)	44 (14%)	10 (14%)	25 (12%)	9 (30%)	0.026
Urate mg/dL, Median (IQR)	315.0 (256.0, 363.0)	315.0 (254.0, 375.0)	318.0 (262.0, 363.0)	274.0 (232.0, 339.0)	0.229
CSF A‐synuclein pg/mL, Median (IQR)	1410.6 (1050.5, 1799.2)	1395.1 (1055.7, 1762.5)	1412.2 (1085.0, 1799.2)	1247.0 (893.3, 1853.0)	0.692
CSF A‐beta 1–42 pg/mL, Median (IQR)	841.1 (613.0, 1125.0)	870.0 (642.9, 1146.0)	842.0 (637.2, 1117.0)	707.0 (535.8, 1128.0)	0.359
CSF A‐beta ≤ 683 pg/mL (A+), *n* (%)	108 (32%)	23 (30%)	69 (30%)	16 (47%)	0.137
CSF t‐tau pg/mL, Median (IQR)	155.2 (124.4, 201.2)	152.6 (125.2, 199.7)	155.3 (125.8, 196.6)	169.8 (115.7, 243.4)	0.735
CSF p‐tau pg/mL, Median (IQR)	12.9 (10.3, 16.8)	12.8 (10.3, 16.6)	12.9 (10.4, 16.7)	13.8 (8.8, 18.3)	0.839
CSF p‐tau ≥ 13 pg/mL (T+), *n* (%)	167 (49%)	35 (45%)	113 (49%)	19 (54%)	0.674
Genetic Risk Score, Median (IQR)	−0.0094 (−0.0120, −0.0072)	−0.0097 (−0.0118, −0.0072)	−0.0093 (−0.0121, −0.0070)	−0.0092 (−0.0120, −0.0076)	0.864

*Note: (Missing)* Serum NfL *n =* 32; Urate *n =* 3; CSF α‐synuclein *n = 6*; CSF A‐beta 1–42 *n = 7*; CSF t‐tau *n = 5*; CSF p‐tau *n = 4*; Genetic Risk Score *n =* 6. (*) Comparisons across NSD stage at baseline used Chi‐Square or Fisher's Exact tests for categorical variables and Kruskal‐Wallis test for continuous variables.

### 3.2 Five Year Longitudinal Clinical and Biologic Characteristics

At 5 years, there were data available for 268 (78%) participants. As expected, MDS‐UPDRS Part I, II, and III scores worsened over time (Table 3). Median [IQR] MDS‐UPDRS Part III medications ON score at year 5 was 24 [15, 31], which represents a 20% increase compared to baseline OFF scores despite 96% of participants being on medication with a median of 598.8 [400.0, 800.0] levodopa equivalent dose (LEDD) use. As a group, median modified S&E was 90 [80, 90] and did not progress. At a group level, median MOCA scores remained above 26. Majority of participants remained cognitively intact (80%) though 17% were characterized as MCI and 4% as dementia. Other non‐motor features remained overall stable except for SCOPA‐AUT scores, which increased over time, with a median [IQR] of 12 [8, 18] and RBDSQ scores with a median [IQR] of 4 [2, 7] at 5 years (Table 3).

**TABLE 3:** Five‐year clinical characteristics.

	Annual visit					
	Baseline (*N* = 345)	Year 1 (*N* = 322)	Year 2 (*N* = 312)	Year 3 (*N* = 304)	Year 4 (*N* = 291)	Year 5 (*N* = 268)
MDS‐UPDRS Part I, Median (IQR)	5.0 (3.0, 7.0)	6.0 (4.0, 9.0)	7.0 (4.0, 10.0)	7.0 (4.5, 11.0)	8.0 (5.0, 13.0)	9.0 (5.0, 13.0)
MDS‐UPDRS Part II, Median (IQR)	5.0 (3.0, 8.0)	7.0 (4.0, 10.0)	7.0 (4.0, 11.0)	8.0 (5.0, 12.0)	9.0 (5.0, 13.0)	9.0 (5.0, 14.0)
MDS‐UPDRS Part III (OFF), Median (IQR)	20.0 (15.0, 26.0)	25.0 (18.0, 32.0)	27.0 (20.0, 35.0)	28.0 (22.0, 38.0)	31.0 (22.0, 38.0)	31.0 (24.0, 39.0)
Missing	0	49	80	85	71	74
MDS‐UPDRS Part III (ON), Median (IQR)	20.0 (15.0, 26.0)	23.0 (16.0, 30.0)	22.0 (15.0, 31.0)	23.0 (15.0, 33.0)	23.0 (14.0, 33.0)	24.0 (15.0, 31.0)
MDS‐UPDRS Part IV, Median (IQR)	—	0.0 (0.0, 0.0)	0.0 (0.0, 0.0)	0.0 (0.0, 0.0)	0.0 (0.0, 3.0)	1.0 (0.0, 4.0)
Missing*	—	15	15	10	4	5
MDS‐UPDRS Total Score (ON), Median (IQR)	31.0 (23.0, 40.0)	37.0 (26.0, 47.0)	36.0 (26.0, 50.0)	38.0 (28.0, 52.0)	41.0 (29.0, 54.0)	42.0 (30.0, 54.5)
On PD Medication, *n* (%)	0	195 (61%)	262 (84%)	281 (92%)	273 (94%)	257 (96%)
LEDD, Median (IQR)	—	242.4 (120.0, 340.0)	300.0 (200.0, 480.0)	400.0 (260.0, 600.0)	500.0 (300.0, 695.0)	598.8 (400.0, 800.0)
Hoehn and Yahr (ON), *n* (%)						
0	0	1 (< 1%)	0	1 (< 1%)	2 (1%)	0
1	153 (44%)	85 (28%)	81 (27%)	64 (23%)	54 (20%)	40 (16%)
2	192 (56%)	215 (70%)	205 (69%)	205 (73%)	202 (74%)	202 (79%)
3	0	5 (2%)	7 (2%)	11 (4%)	12 (4%)	11 (4%)
4	0	1 (< 1%)	2 (1%)	1 (< 1%)	2 (1%)	3 (1%)
5	0	0	0	0	2 (1%)	0
Cognitive Categorization, *n* (%)						
Normal Cognition	71 (91%)	191 (86%)	260 (85%)	241 (80%)	222 (78%)	211 (80%)
Mild Cognitive Impairment (MCI)	7 (9%)	30 (13%)	43 (14%)	58 (19%)	57 (20%)	44 (17%)
Dementia	0	2 (1%)	3 (1%)	4 (1%)	6 (2%)	10 (4%)
Missing	267	99	6	1	6	3
MOCA Total Score, Median (IQR)	28.0 (26.0, 29.0)	27.0 (25.0, 29.0)	27.0 (25.0, 29.0)	27.0 (25.0, 29.0)	27.0 (25.0, 29.0)	27.0 (25.0, 29.0)
SCOPA‐AUT, Median (IQR)	8.0 (5.0, 12.0)	10.0 (6.0, 14.0)	11.0 (7.0, 15.0)	11.0 (8.0, 17.0)	12.0 (8.0, 16.0)	12.0 (8.0, 18.0)
RBD Screening Questionnaire Score, Median (IQR)	3.0 (2.0, 6.0)	4.0 (2.0, 6.0)	4.0 (2.0, 7.0)	4.0 (2.0, 7.0)	4.0 (3.0, 7.0)	4.0 (2.0, 7.0)
Modified Schwab & England, Median (IQR)	90.0 (90.0, 100.0)	90.0 (90.0, 95.0)	90.0 (80.0, 90.0)	90.0 (80.0, 90.0)	90.0 (80.0, 90.0)	90.0 (80.0, 90.0)

*Note:* Variables with missing values less than 10% of the total population at all annual visits are excluded from the table.

* Number missing out of those on symptomatic therapy at the annual visit (eligible to complete MDS‐UPDRS part IV).

**FIGURE 1:**
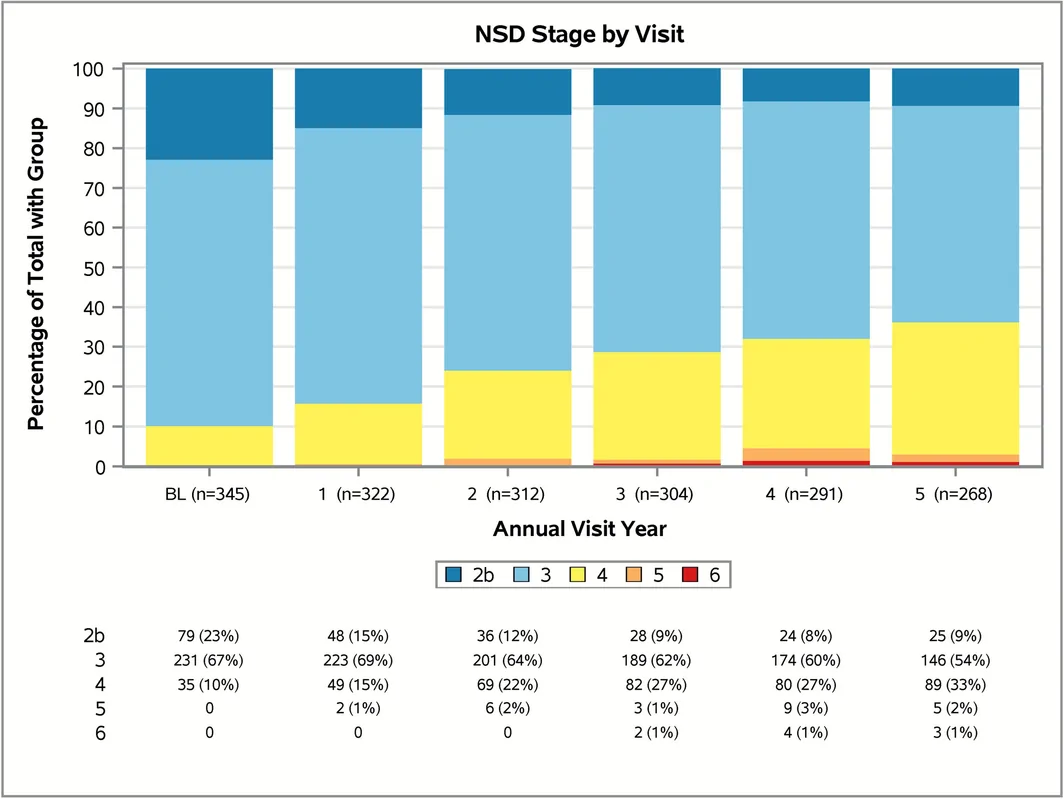
Neuronal Synuclein Stage (NSD) by visit. Shows the percentage of participants in each NSD Stage per annual visit over 5 years. Lower panel data show the total number of participants and percentage in each NSD Stage at each visit.

There was a longitudinal change in the distribution of NSD stages at the cohort level. While at year 5 most of the participants still were NSD Stage 3, the percent of participants in stage 2B reduced from 23 to 9, and the percent of participants in stage 4 increased from 10 to 33, and 3% reached advanced stages 5 and 6 (Figure 1). These stage transitions were present even though the majority (95%) of individuals remained at HY stage 2 or below.

DAT binding data were collected for 4 years. All DAT binding measures worsened over time, with median [IQR] age/sex expected lowest putamen SBR of 0.24 [0.18, 0.28], median [IQR] mean striatum binding 1.00 [0.82, 1.26], median [IQR] mean caudate binding 1.46 [1.18, 1.84], and median [IQR] mean putamen binding 0.53 [0.42, 0.67] at 4 years (Table 4). CSF alpha‐synuclein, amyloid, and tau markers varied over follow‐up, without a consistent longitudinal trajectory. Serum NfL consistently increased over follow‐up (Wald effect estimate [95% CL]: 1.22 [0.64, 1.79]; *p* < 0.0001), although it did not differ by baseline stage (Interaction *p*‐values: Stage 3 vs. 2b: 0.1882; Stage 4 vs. 2b: 0.6623) Figure S2. Longitudinal biofluid biomarker data are summarized in (Table 4 and Figure S2e‐j).

**TABLE 4:** Five‐year imaging and biofluid characteristics.

	Annual visit					
	Baseline (*N* = 345)	Year 1 (*N* = 322)	Year 2 (*N* = 312)	Year 3 (*N* = 304)	Year 4 (*N* = 291)	Year 5 (*N* = 268)
*DAT binding measures*						
Age/Sex‐Expected Lowest Putamen SBR, Median (IQR)	0.31 (0.26, 0.38)	0.28 (0.23, 0.34)	0.26 (0.21, 0.33)	—	0.24 (0.18, 0.28)	—
Mean Striatum Binding, Median (IQR)	1.36 (1.17, 1.62)	1.22 (1.01, 1.45)	1.15 (0.91, 1.36)	—	1.00 (0.82, 1.26)	—
Mean Caudate Binding, Median (IQR)	1.96 (1.67, 2.35)	1.79 (1.46, 2.09)	1.67 (1.33, 2.01)	—	1.46 (1.18, 1.84)	—
Mean Putamen Binding, Median (IQR)	0.79 (0.65, 0.96)	0.66 (0.54, 0.83)	0.62 (0.48, 0.78)	—	0.53 (0.42, 0.67)	—
Missing	0	16	19	—	42	—
*Fluid Biomarkers*						
Serum NFL pg/mL, Median (IQR)	11.4 (8.3, 15.8)	12.2 (9.1, 16.6)	12.7 (9.2, 18.3)	13.5 (10.2, 19.1)	—	15.0 (10.9, 21.9)
Serum NFL ≥ 19.05 pg/mL (N+), *n* (%)	44 (14%)	44 (17%)	59 (22%)	72 (26%)	—	82 (31%)
Missing	32	60	43	23	—	7
Urate mg/dL, Median (IQR)	315.0 (256.0, 363.0)	306.0 (256.0, 363.0)	299.5 (257.0, 357.0)	303.0 (262.0, 357.0)	297.0 (256.0, 354.0)	301.5 (256.0, 363.0)
CSF A‐synuclein pg/mL, Median (IQR)	1410.6 (1050.5, 1799.2)	1292.2 (954.8, 1733.8)	1326.0 (1008.9, 1765.3)	1347.5 (1059.2, 1773.7)	—	—
Missing	6	53	59	111	—	—
CSF A‐beta 1–42 pg/mL, Median (IQR)	841.1 (613.0, 1125.0)	796.0 (591.0, 1065.0)	827.9 (596.4, 1099.5)	842.0 (615.6, 1050.0)	810.3 (605.0, 1038.5)	761.5 (570.7, 1035.1)
CSF A‐beta ≤ 683 pg/mL (A+), *n* (%)	108 (32%)	95 (35%)	89 (35%)	73 (32%)	65 (34%)	68 (43%)
Missing	7	53	60	76	99	108
CSF t‐tau pg/mL, Median (IQR)	155.2 (124.4, 201.2)	153.2 (120.4, 194.4)	154.3 (122.7, 195.5)	155.7 (125.2, 202.7)	153.6 (123.6, 195.4)	145.4 (118.2, 188.0)
Missing	5	52	59	72	99	108
CSF p‐tau pg/mL, Median (IQR)	12.9 (10.3, 16.8)	12.4 (9.6, 16.9)	12.6 (9.9, 16.7)	12.8 (10.2, 17.2)	12.5 (10.3, 16.0)	12.3 (9.6, 15.8)
CSF p‐tau ≥ 13 pg/mL (T+), *n* (%)	167 (49%)	124 (46%)	119 (47%)	110 (47%)	85 (44%)	67 (42%)
Missing	4	52	59	72	99	108

*Note:* Variables with missing values less than 10% of the total population at all annual visits are excluded from the table.

### 3.3 Five‐Year Outcomes by Baseline NSD Stage

**FIGURE 2:**
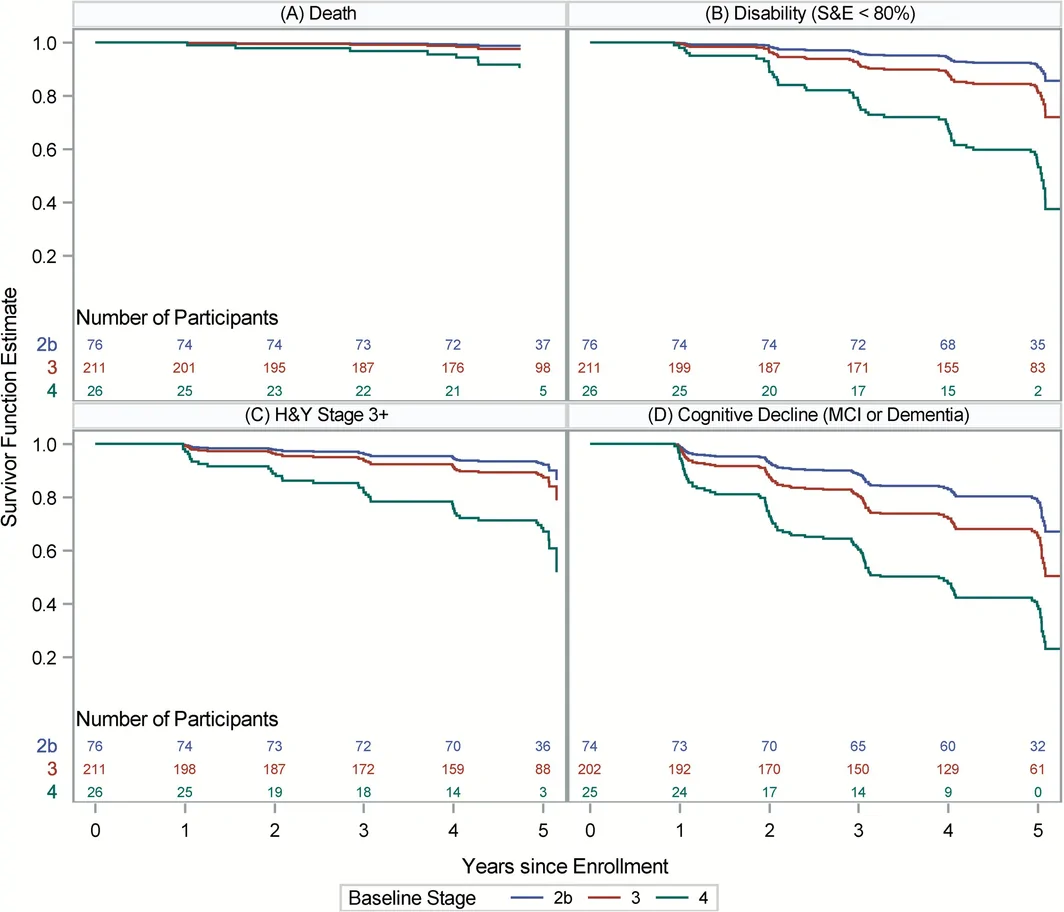
Time to death, disability, postural instability, and cognitive decline by NSD Stage, adjusted by age, sex, and education.

**FIGURE 3:**
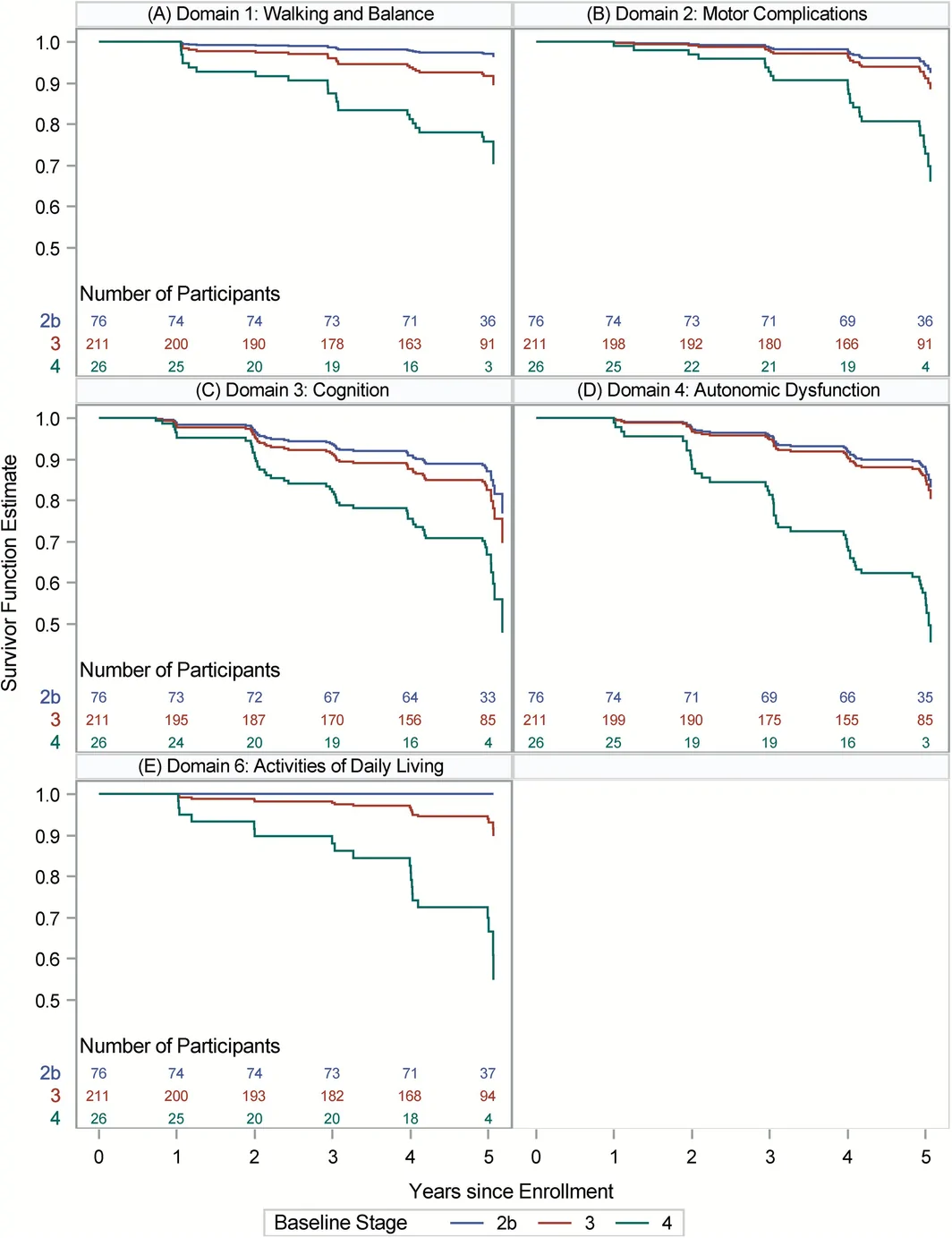
Time to milestone domains, by NSD Stage, adjusted by age, sex, and education.

Figures 2 and 3 summarize time to reaching clinically meaningful milestones of functional decline presented by baseline NSD stage. 32 participants were excluded from the survival analyses due to reaching an event of interest at baseline. Of the 313 participants included, 76 were in stage 2b, 211 were in stage 3, and 26 were in stage 4 at baseline. 9 participants died over 5 years of observation (1 was in stage 2b at baseline, 5 in stage 3 at baseline, and 3 in stage 4 at baseline). After adjusting for age, sex, and education, there were no significant differences in the event rate of clinically meaningful milestones between baseline stages 3 and 2b. Participants in stage 4 at baseline, however, had a significantly higher rate of reaching disability, H&Y ≥ 3, cognitive decline, the walking and balance milestone, and the autonomic dysfunction milestone compared to those in stage 2b at baseline (Table S3).

## 4 Discussion

We present a detailed description of five‐year clinical and biological data in a cohort of individuals with early sPD at enrollment, all of whom had a confirmed biomarker of αSyn pathology. Our observations offer insights that could inform future treatment trials design.

While longitudinal data on the rate of progression of key clinical measures are consistent with previous reports from non‐biomarker defined cohorts, these data are of value for the design and power estimation of future therapeutic studies targeting a biomarker defined population. These data highlight the challenges of identifying responsive outcome measures in the early stage of disease [36]. Despite being S+ and being recruited with the same inclusion criteria into PPMI, participants exhibited significant heterogeneity in functional status at baseline. While most participants were in NSD Stage 3, defined by a slight level of functional impairment, 23% were in NSD Stage 2B defined by lack of functional impairment, and importantly, 10% were in NSD Stage 4. There was a stage‐dependent difference in baseline clinical characteristics that were not part of the staging anchors. Heterogeneity of the population is one of the major limitations in the ability to have a reliable readout in clinical trials [36]. As our study shows, traditional clinical trial enrollment criteria, which often rely on time since diagnosis or broad clinical definitions, fail to account for this variability, potentially diluting treatment effects and hindering the identification of disease‐modifying therapies [37]. A more refined approach could involve stratifying trial populations using biological and functional markers [38, 39]. Stage‐based recruitment might provide a tool to reduce such heterogeneity. That is specifically relevant for individuals in NSD Stage 4. While they represent a small percent of the overall cohort, their baseline clinical and biological characteristics and, importantly, progression curves are substantially different. Depending on the objectives, interventional studies might exclude these individuals as fast progressors to reduce population heterogeneity or, on the contrary, specifically seek NSD Stage 4 individuals to assess the impact of treatment on the more “malignant” disease phenotype [40, 41].

It is not surprising that individuals in more advanced baseline stages progress faster on the functional measures of decline. However, the field of PD has been searching for operational definitions of disease subtypes [42, 43, 44, 45, 46, 47]. Recently, the Movement Disorder Society Task Force on Subtyping published their critical evaluation on current subtyping systems and concluded that the subtyping studies undertaken to date have significant methodologic shortcomings, with questionable clinical applicability and with unknown biological relevance [47]. In this context, our findings suggest that NSD staging offers one potential framework to operationalize subtyping by integrating motor, cognitive, and other non‐motor functional impairment, an approach that may ultimately help refine research cohort selection and optimize trial desgin [14]. Moving toward biomarker‐defined cohorts will be crucial for the success of future disease‐modifying trials in PD, ensuring that treatment effects are evaluated in a homogeneous population.

The goal of the field is to understand the biological drivers of disease heterogeneity. Conducting such studies in individuals with unifying αSyn pathology is the first step. Our analysis of the biomarkers panel did not “unlock the code” but shed some light. There were stage dependent differences in the DAT measures, which increases confidence in the fact that stages reflect an increasing degree of biological burden of the disease at least in the domain of dopaminergic deficit. Our analysis showed that striatal DAT binding values were significantly lower at baseline and remained lower throughout follow‐up in NSD Stage 4 participants. The association between reduced DAT binding and worse longitudinal motor outcomes has been reported in prior, smaller studies, though findings have been inconsistent. While quantitative DAT is a reliable measure of dopaminergic dysfunction that is widely accepted as an enrichment biomarker in PD clinical trials, it has failed to demonstrate sensitivity to change in PD interventional trials [48, 49]. One of the limitations might have been an αSyn heterogeneous population. These data can be used for the design of the early phase studies using DAT as a proof‐of‐concept biomarker.

We also have explored biomarkers of amyloid and tau co‐pathology but failed to demonstrate baseline stage‐based separation of the tested measures. There was not an observed difference in longitudinal change in these measures at the group level either. This might reflect early stages of neurodegeneration in this cohort in which the presence of co‐pathology is limited; alternatively, the panel of biomarkers tested may be insufficient. PPMI is currently testing a comprehensive panel of biomarkers of neurodegeneration and the results will be reported once available. A critical avenue for future research is the development of a quantitative biomarker for αSyn pathology and its correlation with clinical progression. While biomarkers such as A*β* 1–42, tau, and p‐tau have been explored in PD, particularly for their association with cognitive decline and motor outcomes, findings remain inconsistent [39, 40, 41].

Over 5 years of follow‐up, we observed an overall slow disease progression, with only a limited number of participants reaching key milestones such as loss of independence, postural instability, cognitive impairment, or death. This may, in part, be due to the exclusion of S− participants, who are hypothesized to exhibit a different pathological process. While most individuals with sPD are expected to be S+, even a small subset with alternative pathology yet a similar phenotype may significantly influence data interpretation regarding disease progression. In the PPMI cohort, 93% of individuals with sPD are S + [6]. A separate analysis of the S− sPD phenotype is currently underway and will be reported in a separate publication.

There was a time dependent change in the proportion of participants in Stage 2B. As expected, the percentage of individuals in NSD Stage 2B dropped from 23 to 9 by year 5, and the percentage of participants in NSD Stage 4 increased from 10 to 33. These changes occurred while majority of individuals remained at HY stage 2. These data support a wider dynamic range of NSD staging. We have recently explored the impact of dopaminergic therapy on the stability of staging and demonstrated minimal effect in early stages (NSD Stage 3 or below) [50]. These findings may imply that enrolling individuals on stable medications using NSD staging might be a feasible approach for clinical trials design. While stage transition might become a relevant endpoint for disease modification trials, currently it is premature, and more data need to be collected to validate the performance of this measure.

Importantly, we emphasize that the NSD staging system is intended for research purposes currently. It has not been validated for clinical decision‐making. As such, we present NSD staging as a research tool to better characterize study populations, rather than as a system ready for routine clinical use or for guiding clinical management.

This study has limitations. Generalizability might be limited by the characteristics of the PPMI cohort. The PPMI cohort is younger and medication naïve at baseline. Nonetheless, PPMI remains comparable to other clinical trial populations, making our findings highly relevant for future trials design. We acknowledge the limited racial and ethnic diversity in PPMI, and efforts are underway to improve inclusivity in participant recruitment.

Additionally, NSD criteria require confirmation of αSyn pathology currently assessed by CSF. Expanding assessments to less invasive biofluid measures would enhance broader applicability. Despite this limitation, our findings reinforce the potential of NSD‐ISS for clinical trials selection.

Retention in observational long‐term studies is a universal challenge. We report participants' retention within the PPMI cohort comparable to prior longitudinal cohorts [8, 9, 40, 41]; this is of importance considering the duration and substantial scope of ascertainments.

The event‐based model assumes a uniform progression sequence among S+ participants, which does not fully capture the clinical heterogeneity observed in PD. Future refinements to this model will aim to account for variations in the timing and sequence of disease milestones.

Although PPMI prioritizes biological characterization, current data lack a validated quantitative biomarker for NSD progression. At this point, αSyn‐SAA in CSF is reported only as a qualitative (positive/negative) determination based on replicate concordance. While outside the scope of the current analysis, developing quantitative biomarkers of αSyn pathology including but not limited to incorporating SAA kinetic measures (time to threshold, time to maximum fluorescence) is part of ongoing efforts. PPMI also aims to integrate novel biomarkers, including omics‐based approaches, to further delineate disease biology.

## 5 Conclusion

This study presents 5‐year longitudinal data on deeply phenotypically and biologically characterized S+ sPD participants. Our findings highlight substantial heterogeneity among “early” PD participants, despite uniform enrollment criteria. Baseline NSD stage strongly predicted progression, underscoring the need to shift clinical trial design from arbitrary time‐since‐diagnosis criteria toward biologically and functionally precise participant selection. Biological underpinnings of baseline and progression heterogeneity are the focus of ongoing research.

## Author Contributions

Paulina Gonzalez‐Latapi, Caroline Gochanour, Hyunkeun Cho, Seung Ho. Choi, Christopher Coffey, Caroline Tanner, Kathleen L. Poston, Andrew Siderowf, Kenneth Marek and Tanya Simuni contributed to the conception and design of the study; all authors contributed to the acquisition and analysis of data; Paulina Gonzalez‐Latapi, Caroline Gochanour, Seung Ho Choi, Caroline Tanner, Andrew Siderowf, Kenneth Marek, Tanya Simuni contributed to drafting the text or preparing the figures. Comprehensive The Parkinson's Progression Markers Initiative author list is included in Supporting Informations.

## Funding

The authors have nothing to report.

## Conflicts of Interest

Paulina Gonzalez‐Latapi declares grant from The Parkinson's Progression Markers Initiative supported by The Michael J. Fox Foundation. She has also received research grants from The Michael J. Fox Foundation and the Parkinson's Foundation. Caroline Gochanour declares employment for The Michael J. Fox Foundation. Hyunkeun Cho declares travel grants from The Michael J. Fox Foundation. Seung Ho Choi declares travel grants from The Michael J. Fox Foundation. Chelsea Caspell‐Garcia reports no disclosures. Caroline Gochanour declares grants from The Michael J. Fox Foundation and NIH/NINDS. Michael Brumm declares travel grants from The Michael J. Fox Foundation. David‐Erick Lafontant declares travel grants from The Michael J. Fox Foundation. Yuge Xiao declares employment for and travel grants from The Michael J. Fox Foundation. Caroline Tanner declares consultancies for CNS Ratings, Australian Parkinson's Mission, Biogen, Evidera, Cadent (data safety monitoring board), Adamas (steering committee), Biogen (via the Parkinson Study Group steering committee), Kyowa Kirin (advisory board), Lundbeck (advisory board), Jazz/Cavion (steering committee), Acorda (advisory board), Bial (DMC) and Genentech. Caroline Tanner also declares grant support to her institution from The Michael J. Fox Foundation, National Institute of Health, Gateway LLC, Department of Defense, Roche Genentech, Biogen, Parkinson Foundation and Marcus Program in Precision Medicine. Caroline Tanner declares membership on the npj Parkinson's Disease Editorial Board. CV declares grants from NIH/NINDS. Karl Kieburtz is an employee of the University of Rochester, that receives funding from the Michael J Fox Foundation, and is an employee of Clintrex Research LLC, a division of Tox Strategies, who receive funding from commercial clients. Karl Kieburtz has equity interests in Tox Strategies, Safe Therapeutics, Inhibikase, Photopharmics, Biohaven and Hoover Brown LLC. LC declares grants to her institution from Biogen (clinical trial funding), MJFF, UPMC Competitive Medical Research Fund, National Institutes of Health, and University of Pittsburgh; grant and travel support from MJFF; royalties from Wolters Kluwel (for authorship); and in‐kind donation by Advanced Brain Monitoring of equipment for research study to her institution. KP declares consultancies for Curasen; was on a scientific advisory board for Curasen and Amprion; honoraria from invited scientific presentations to universities and professional societies not exceeding $5000 per year from California Congress of Clinical Neurology, California Neurological Society, and Johns Hopkins University; and patents or patent applications numbers 17/314,979 and 63/377,293. KP also declares grants to her institution (Stanford University School of Medicine) from NIH/NINDS NS115114, NS062684, NS075097, NIH/NIA U19 AG065156, P30 AG066515, The Michael J. Fox Foundation, Lewy Body Dementia Association, Alzheimer's Drug Discovery Foundation, Sue Berghoff. Andrew Siderowf declares consultancies for SPARC Therapeutics, Capsida Therapeutics and Parkinson Study Group; honoraria from Bial; grants from The Michael J. Fox Foundation (member of PPMI Steering Committee); and participation on board at Wave Life Sciences, Inhibikase, Prevail, Huntington Study Group and Massachusetts General Hospital. Kenneth Marek declares support to his institution (Institute for Neurodegenerative Disorders) from The Michael J. Fox Foundation. Kenneth Marek also declares consultancies for Invicro, The Michael J. Fox Foundation, Roche, Calico, Coave, Neuron23, Orbimed, Biohaven, Anofi, Koneksa, Merck, Lilly, Inhibikase, Neuramedy, IRLabs and Prothena and participates on DSMB at Biohaven. Tanya Simuni declares consultancies for AcureX, Adamas, AskBio, Amneal, Blue Rock Therapeutics, Critical Path for Parkinson's Consortium, Denali, The Michael J. Fox Foundation, Neuroderm, Roche, Sanofi, Sinopia, Takeda, and Vanqua Bio; on advisory boards for AcureX, Adamas, AskBio, Biohaven, Denali, GAIN, Neuron23 and Roche; on scientific advisory boards for Koneksa, Neuroderm, Sanofi and UCB; and received research funding from Amneal, Biogen, Roche, Neuroderm, Sanofi, Prevail and UCB and an investigator for NINDS, MJFF, Parkinson's Foundation.

## Supporting information


**Figure S1:** Participant Flowchart
Caption: gPD (genetic PD); NSD (Neuronal Synuclein Disease); PD (Parkinson's Disease); sPD (sporadic PD).

**Figure S2a:** Age/Sex‐Expected Lowest Putamen SBR over time.

**Figure S2b:** Mean Striatum Over Time.
DAT binding over time.

**Figure 2c** Mean Caudate Over Time.
DAT binding over time.

**Figure S2d:** Mean Putamen Over Time.
DAT binding over time.

**Figure S2e:** Serum NfL over time.
NfL (Neurofilament ligand).

**Figure S2f:** Urate over time.
No caption.

**Figure S2g:** CSF‐A‐synuclein over time.
A‐synuclein (alpha‐synuclein); CSF (cerebrospinal fluid).

**Figure S2h:** CSF A‐beta 1–42 over time.
A‐beta (amyloid beta); CSF (cerebrospinal fluid).

**Figure S2i:** CSF t‐tau over time.
CSF (cerebrospinal fluid); t‐tau (total tau).

**Figure S2j:** CSF p‐tau over time.
CSF (cerebrospinal fluid); p‐tau (phosphorylated tau).

**Data S1:** Tables S1‐S4.

## Data Availability

Data used in the preparation of this article were obtained on June 24, 2024 from the Parkinson's Progression Markers Initiative (PPMI) database (www.ppmi‐info.org/access‐data‐specimens/download‐data), RRID:SCR 006431. For up‐to‐date information on the study, visit www.ppmi‐info.org. This analysis was conducted by the PPMI Statistics Core and used actual dates of activity for participants, a restricted data element not available to public users of PPMI data. Statistical analysis codes used to perform the analyses in this article are shared on Zenodo [10.5281/zenodo.11660808].
