# Supplementary material for: Five‐Year Disease Progression in Synuclein Seeding Positive Sporadic Parkinson's Disease
Source: Ann Clin Transl Neurol. 2026 Mar 10;13(9):1791–806. doi: 10.1002/acn3.70323 (PMC13393511; doi:10.1002/acn3.70323)
Supplement: Supplementary file 1 [file ACN3-13-1791-s007.pdf]

Supplementary Table 1. NSD Staging Criteria*

	Biologic anchors			Anchors of clinical signs or symptoms (stages 2A and 2B) and functional impairment (stages 3-6)[1, 2]	
**Stage**	**S**	**D** [a]	**G**	**Domain**	**Anchor(s)**
Stage 0	-	-	*SNCA* [b]	—	—
Stage 1A	+	-	±	(1) Cognitive<br>(2) Motor<br>(3) Other non-motor	(1) MDS-UPDRS item 1.1 = 0; and<br>(2a) Does not have subthreshold parkinsonism [c]; and (2b) is not on PD meds; and<br>(3a) Does not have RBD; and (3b) is not hyposmic [d]
Stage 1B	+	+	±		
Stage 2A	+	-	±	(1) Cognitive<br>(2) Motor<br>(3) Other non-motor	(1) Item 1.1 = 1 AND MoCA ≥ 25; or<br>(2a) Has subthreshold parkinsonism [c]; or (2b) is on PD meds [e]; or<br>(3a) Has RBD; or (3b) is hyposmic [d]
Stage 2B	+	+	±		
Stage 3	+	+	±	(1) Cognitive<br>(2) Motor	(1a) Item 1.1 = 1 AND MoCA ≤ 24; or (1b) Item 1.1 = 2 AND MoCA ≥ 25; or<br>(2) MDS-UPDRS-II = 3-13 AND either subthreshold parkinsonism [c] or PD meds [e]
Stage 4	+	+	±	(1) Cognitive<br>(2) Motor<br>(3) Other non-motor	(1a) Item 1.1 = 2 and MoCA ≤ 24; or (1b) item 1.1 = 3 AND MoCA ≥ 25; or<br>(2) MDS-UPDRS-II = 14-26; or<br>(3) MDS-UPDRS-I (excluding item 1.1) = 13-24 [f]
Stage 5	+	+	±	(1) Cognitive<br>(2) Motor<br>(3) Other non-motor	(1a) Item 1.1 = 3 AND MoCA ≤ 24; or (1b) item 1.1 = 4 AND MoCA ≥ 25; or<br>(2) MDS-UPDRS-II = 27-39; or<br>(3) MDS-UPDRS-I (excluding item 1.1) = 25-36
Stage 6	+	+	±	(1) Cognitive<br>(2) Motor<br>(3) Other non-motor	(1) Item 1.1 = 4 AND MoCA ≤ 24; or<br>(2) MDS-UPDRS-II ≥ 40; or<br>(3) MDS-UPDRS-I (excluding item 1.1) ≥ 37

* Adapted from: Simuni T, Chahine LM, Poston K, et al. A biological definition of neuronal alpha-synuclein disease: towards an integrated staging system for research. Lancet Neurol. 2024 Feb;23(2):178-90.

[1] Presence of qualifying signs/ symptoms in any single domain qualifies for stage 2 but individuals can have combination in all 3 domains.

[2] Presence of qualifying functional impairment in any single domain qualifies for stage 3-6 but individuals can have combination in all 3 domains.

[a] D positivity defined as < 75% age/sex-expected lowest putamen SBR.

[b] Only fully penetrant pathogenic *SNCA* variants qualify for Stage 0.

[c] Subthreshold parkinsonism defined as MDS-UPDRS-III ≥ 5 excluding postural and action tremor.

[d] Hyposmia defined as UPSIT percentile ≤ 15 (age and sex adjusted).

[e] Presence of PD medication is determined by the MDS-UPDRS Item III question 3a.

[f] MDS-UPDRS-I (excluding item 1.1) ≥ 13 is sufficient for stage 4 provided that stage 2 criteria are met.

Supplementary Table 2: Milestone criteria*

Progression milestone	Assessment(s)	Criteria
**Domain 1: Walking and balance**		
Walking and balance	MDS-UPDRS item 2.12	Response ≥3
Freezing	MDS-UPDRS item 2.13	Response ≥3
Gait	MDS-UPDRS item 3.10	Response ≥3 (ON or OFF)
Freezing of gait	MDS-UPDRS item 3.11	Response = 4 (ON or OFF)
Postural instability	MDS-UPDRS item 3.12	Response ≥3 (ON or OFF)
Hoehn and Yahr stage	Hoehn and Yahr Stage	Response ≥4 (ON or OFF)
**Domain 2: Motor complications**		
Dyskinesias	MDS-UPDRS items 4.1 and 4.2	Response ≥3 (on *both* items)
Fluctuations (functional impact)	MDS-UPDRS item 4.4	Response ≥3
Fluctuations (complexity)	MDS-UPDRS item 4.5	Response ≥3
**Domain 3: Cognition**		
Cognitive impairment (MoCA)[*]	MoCA	Score<21
Cognitive impairment (MDS-UPDRS)	MDS-UPDRS item 1.1	Response ≥3
Hallucinations	MDS-UPDRS item 1.2	Response ≥3
Apathy	MDS-UPDRS item 1.5	Response ≥3
Dementia (clinical diagnosis)[*]	Site investigator assessment	PDD (per Investigator)
Dementia (composite)[*]	(1) Cognitive testing	Impairment [†] on ≥2 cognitive domains; and
	(2) Site investigator assessment	Functional impairment (per investigator)
**Domain 4: Autonomic dysfunction**		
Urinary incontinence[**]	(1) MDS-UPDRS item 1.10	Response ≥3; and
(2) SCOPA-AUT items 8 and 9	Response ≥2 (on *either* item)	
Orthostatic hypotension[**]	(1) SCOPA-AUT item 15	Response ≥2; and
(2) Systolic blood pressure	Change of ≥20 mm Hg (sitting to standing); and	
(3) Diastolic blood pressure	Change of ≥10 mm Hg (sitting to standing)	
Syncope (MDS-UPDRS)	MDS-UPDRS item 1.12	Response = 4
Syncope (SCOPA-AUT)[**]	SCOPA-AUT item 16	Response ≥1
**Domain 5: Functional dependence**		
Schwab &England	Schwab &England	Response<80
**Domain 6: Activities of daily living**		
Choking	MDS-UPDRS item 2.3	Response ≥3
Eating	MDS-UPDRS item 2.4	Response ≥3
Dressing	MDS-UPDRS item 2.5	Response ≥3
Hygiene	MDS-UPDRS item 2.6	Response ≥3
Speech	MDS-UPDRS item 3.1	Response ≥3 (ON or OFF)

* Adapted from: Brumm MC, Siderowf A, Simuni T, et al. Parkinson's Progression Markers Initiative: A Milestone-Based Strategy to Monitor Parkinson's Disease Progression. J Parkinsons Dis. 2023;13(6):899-916.
Unless otherwise specified, milestones were assessed at 3, 6, 9, 12, 18, 24, 30, 36, 42, 48, 54, and 60 months.
[*]Assessed at annual visits only. [**]Assessed at 6 months and annual visits only. [†]Impairment defined as a test score ≥1.5 standard deviations below the standardized mean score. MDS-UPDRS, Movement Disorder Society Unified Parkinson's Disease Rating Scale; MoCA, Montreal Cognitive Assessment; PDD, Parkinson's Disease Dementia; SCOPA-AUT, Scales for Outcomes in Parkinson's Disease-Autonomic.

Supplementary Table 3. Cox Regression Model Results

Outcome	Hazard Ratio (95% Wald CL)	Hazard Ratio P-value
**Death**		
BL Stage 3 vs 2b	1.89 (0.22, 16.30)	0.5619
BL Stage 4 vs 2b	7.07 (0.62, 80.67)	0.1153
**Disability (S&E < 80%)**		
BL Stage 3 vs 2b	2.15 (0.95, 4.83)	0.0655
BL Stage 4 vs 2b	6.73 (2.51, 18.05)	0.0001
**H&Y ≥ 3**		
BL Stage 3 vs 2b	1.70 (0.65, 4.47)	0.2808
BL Stage 4 vs 2b	5.64 (1.86, 17.10)	0.0022
**Cognitive Decline**		
BL Stage 3 vs 2b	1.84 (1.07, 3.18)	0.0281
BL Stage 4 vs 2b	4.62 (2.27, 9.44)	<0.0001
**Domain 1: Walking & Balance**		
BL Stage 3 vs 2b	2.96 (0.68, 12.93)	0.1486
BL Stage 4 vs 2b	9.86 (1.97, 49.41)	0.0054
**Domain 2: Motor Complications**		
BL Stage 3 vs 2b	1.56 (0.52, 4.69)	0.4255
BL Stage 4 vs 2b	5.38 (1.50, 19.29)	0.0098
**Domain 3: Cognition**		
BL Stage 3 vs 2b	1.40 (0.67, 2.91)	0.3725
BL Stage 4 vs 2b	3.05 (1.16, 7.98)	0.0234
**Domain 4: Autonomic Dysfunction**		
BL Stage 3 vs 2b	1.20 (0.56, 2.55)	0.6365
BL Stage 4 vs 2b	4.86 (1.88, 12.58)	0.0011

Supplementary Table 4: List of Acronyms and abbreviations

Acronym	Definition
αSyn	Alpha-synuclein
Aβ 1–42	Amyloid-beta 1–42
ATN(PD)	Amyloid/Tau/Neurodegeneration framework for Parkinson’s Disease
CSF	Cerebrospinal fluid
CSF-SAA	Cerebrospinal fluid synuclein seed amplification assay
DAT	Dopamine transporter
D+ / D–	Dopaminergic dysfunction present / absent
GDS	Geriatric Depression Scale
H&Y	Hoehn and Yahr scale
LEDD	Levodopa Equivalent Daily Dose
MCI	Mild cognitive impairment
MDS-UPDRS	Movement Disorder Society – Unified Parkinson’s Disease Rating Scale
MoCA	Montreal Cognitive Assessment
NfL	Neurofilament light chain
NSD	Neuronal Synuclein Disease
NSD-ISS	Neuronal Synuclein Disease – Integrated Staging System
PDD	Parkinson’s Disease Dementia
RBDSQ	REM Sleep Behavior Disorder Screening Questionnaire
S+ / S–	Synuclein seed amplification assay positive / negative
S&E	Schwab & England Activities of Daily Living Scale
SAA	Seed amplification assay
SBR	Striatal binding ratio
SCOPA-AUT	Scales for Outcomes in Parkinson’s Disease–Autonomic Symptoms
SNCA	Alpha-synuclein gene
UPSIT	University of Pennsylvania Smell Identification Test

PPMI STUDY TEAMS/CORES/COLLABORATORS FOR PUBLICATIONS

Executive Steering Committee:
Kenneth Marek, MD[1] (Principal Investigator); Caroline Tanner, MD, PhD[9]; Tanya Simuni, MD[3]; Andrew Siderowf, MD, MSCE[12]; Douglas Galasko, MD[27]; Lana Chahine, MD[39]; Christopher Coffey, PhD[4]; Kalpana Merchant, PhD[59]; Kathleen Poston, MD[38]; Roseanne Dobkin, PhD[41]; Tatiana Foroud, PhD[15]; Brit Mollenhauer, MD[8]; Dan Weintraub, MD[12]; Ethan Brown, MD[9]; Karl Kieburtz, MD, MPH[23]; Mark Frasier, PhD[6]; Todd Sherer, PhD[6]; Sohini Chowdhury, MA[6]; Roy Alcalay, MD[35] and Aleksandar Videnovic, MD[45]

Steering Committee:
Duygu Tosun-Turgut, PhD[9]; Werner Poewe, MD[7]; Susan Bressman, MD[14]; Jan Hammer[15]; Raymond James, RN[22]; Ekemini Riley, PhD[40]; John Seibyl, MD[1]; Leslie Shaw, PhD[12]; David Standaert, MD, PhD[18]; Sneha Mantri, MD, MS[60]; Nabila Dahodwala, MD[12]; Michael Schwarzschild[45]; Connie Marras[43]; Hubert Fernandez, MD[25]; Ira Shoulson, MD[23]; Helen Rowbotham[2]; Paola Casalin[11] and Claudia Trenkwalder, MD[8]

Michael J. Fox Foundation (Sponsor): Todd Sherer, PhD; Sohini Chowdhury, MA; Mark Frasier, PhD; Jamie Eberling, PhD; Katie Kopil, PhD; Alyssa O'Grady; Maggie McGuire Kuhl; Leslie Kirsch, EdD and Tawny Willson, MBS

Study Cores, Committees and Related Studies: ***(Include as applicable to the paper)***
Project Management Core: Emily Flagg, BA[1]
Site Management Core: Tanya Simuni, MD[3]; Bridget McMahon, BS[1]
Strategy and Technical Operations: Craig Stanley, PhD[1]; Kim Fabrizio, BA[1]
Data Management Core: Dixie Ecklund, MBA, MSN[4]; Trevis Huff, BSE[4]
Screening Core: Tatiana Foroud, PhD[15]; Laura Heathers, BA[15]; Christopher Hobbick, BSCE[15]; Gena Antonopoulos, BSN[15]
Imaging Core: John Seibyl, MD[1]; Kathleen Poston, MD[38]
Statistics Core: Christopher Coffey, PhD[4]; Chelsea Caspell-Garcia, MS[4]; Michael Brumm, MS[4]
Bioinformatics Core: Arthur Toga, PhD[10]; Karen Crawford, MLIS[10]
Biorepository Core: Tatiana Foroud, PhD[15]; Jan Hamer, BS[15]
Biologics Review Committee: Brit Mollenhauer[8]; Doug Galasko[27]; Kalpana Merchant[59]
Genetics Core: Andrew Singleton, PhD[13]
Pathology Core: Tatiana Foroud, PhD[15]; Thomas Montine, MD, PhD[38]
Found: Caroline Tanner, MD PhD[9]
PPMI Online: Carlie Tanner, MD PhD[9]; Ethan Brown, MD[9]; Lana Chahine, MD[39]; Roseann Dobkin, PhD[41]; Monica Korell, MPH[9]

Site Investigators:

Charles Adler, PhD[49]; Roy Alcalay, MD[35]; Amy Amara, PhD[50]; Paolo Barone, PhD[30]; Bastiaan Bloem, PhD[58] Susan Bressman, MD[14]; Kathrin Brockmann, MD[26]; Norbert Brüggemann, MD[57]; Lana Chahine, MD[39]; Kelvin Chou, MD[42]; Nabila Dahodwala, MD[12]; Alberto Espay, MD[32]; Stewart Factor, DO[16]; Hubert Fernandez, MD[25]; Michelle Fullard, MD[50]; Douglas Galasko, MD[27]; Robert Hauser, MD[19]; Penelope Hogarth, MD[17]; Shu-Ching Hu, PhD[21]; Michele Hu, PhD[56]; Stuart Isaacson, MD[31]; Christine Klein, MD[57]; Rejko Krueger, MD[2]; Mark Lew, MD[47]; Zoltan Mari, MD[54]; Connie Marras, PhD[43]; Maria Jose Martí, PhD[33]; Nikolaus McFarland, PhD[52]; Tiago Mestre, PhD[44]; Brit Mollenhauer, MD[8]; Emile Moukheiber, MD[28]; Alastair Noyce, PhD[61] Wolfgang Oertel, PhD[62]; Njideka Okubadejo, MD[63]; Sarah O'Shea, MD[37]; Rajesh Pahwa, MD[46]; Nicola Pavese, PhD[55]; Werner Poewe, MD[7]; Ron Postuma, MD[53]; Giulietta Riboldi, MD[51]; Lauren Ruffrage, MS[18]; Javier Ruiz Martinez, PhD[34]; David Russell, PhD[1]; Marie H Saint-Hilaire, MD[22]; Neil Santos, BS[49]; Wesley Schlett[45]; Ruth Schneider, MD[23]; Holly Shill, MD[48]; David Shprecher, DO[24]; Tanya Simuni, MD[3]; David Standaert, PhD[18]; Leonidas Stefanis, PhD[36]; Yen Tai, PhD[29]; Caroline Tanner, PhD[9]; Arjun Tarakad, MD[20]; Eduardo Tolosa PhD[33] and Aleksandar Videnovic, MD[45]

Coordinators:

Susan Ainscough, BA[30]; Courtney Blair, MA[18]; Erica Botting[19]; Isabella Chung, BS[54]; Kelly Clark[24]; Ioana Croitoru[34]; Kelly DeLano, MS[32]; Iris Egner, PhD[7]; Fahrial Esha, BS[51]; May Eshel, MSc[35]; Frank Ferrari, BS[42]; Victoria Kate Foster[55]; Alicia Garrido, MD[33]; Madita Grümmer[57]; Bethzaida Herrera[48]; Ella Hilt[26]; Chloe Huntzinger, BA[50]; Raymond James, BS[22]; Farah Kausar, PhD[9]; Christos Koros, MD, PhD[36]; Yara Krasowski, MSc[58]; Dustin Le, BS[17]; Ying Liu, MD[50]; Taina M. Marques, PhD[2]; Helen Mejia Santana, MA[37]; Sherri Mosovsky, MPH[39]; Jennifer Mule, BS[25]; Philip Ng, BS[43]; Lauren O'Brien[46]; Abiola Ogunleye, PGDip[29]; Oluwadamilola Ojo, MD[63]; Obi Onyinanya, BS[28]; Lisbeth Pennente, BA[31]; Romina Perrotti[53]; Michael Pileggi, MS[53]; Ashwini Ramachandran, MSc[12]; Deborah Raymond, MS[14]; Jamil Razzaque, MS[56]; Shawna Reddie, BA[44]; Kori Ribb, BSN,[28]; Kyle Rizer, BA[52]; Janelle Rodriguez, BS[27]; Stephanie Roman, HS[1]; Clarissa Sanchez, MPH[20]; Cristina Simonet, PhD[29]; Anisha Singh, BS[23]; Elisabeth Sittig, RN[62]; Barbara Sommerfeld MSN[16]; Angela Stovall, BS[42]; Bobbie Stubbeman, BS[32]; Alejandra Valenzuela, BS[47]; Catherine Wandell, BS[21]; Diana Willeke[8]; Karen Williams, BA[3] and Dilinuer Wubuli, MB[43]

Partners Scientific Advisory Board (Acknowledgement)

Funding: PPMI – a public-private partnership – is funded by the Michael J. Fox Foundation for Parkinson's Research and funding partners, including 4D Pharma, Abbvie, AcureX, Allergan, Amathus Therapeutics, Aligning Science Across Parkinson's, AskBio, Avid Radiopharmaceuticals, BIAL, BioArctic, Biogen, Biohaven, BioLegend, BlueRock Therapeutics, Bristol-Myers Squibb, Calico Labs, Capsida Biotherapeutics, Celgene, Cerevel Therapeutics, Coave Therapeutics, DaCapo Brainscience, Denali, Edmond J. Safra Foundation, Eli Lilly, Gain Therapeutics, GE HealthCare, Genentech, GSK, Golub Capital, Handl Therapeutics, Insitro, Jazz Pharmaceuticals, Johnson & Johnson Innovative Medicine, Lundbeck, Merck, Meso Scale Discovery, Mission Therapeutics, Neurocrine Biosciences, Neuron23, Neuropore, Pfizer, Piramal, Prevail Therapeutics, Roche, Sanofi, Servier, Sun Pharma Advanced Research Company, Takeda, Teva, UCB, Vanqua Bio, Verily, Voyager Therapeutics, the Weston Family Foundation and Yumanity Therapeutics.

1 Institute for Neurodegenerative Disorders, New Haven, CT
2 University of Luxembourg, Luxembourg
3 Northwestern University, Chicago, IL
4 University of Iowa, Iowa City, IA
5 VectivBio AG
6 The Michael J. Fox Foundation for Parkinson's Research, New York, NY
7 Innsbruck Medical University, Innsbruck, Austria
8 Paracelsus-Elena Klinik, Kassel, Germany
9 University of California, San Francisco, CA
10 Laboratory of Neuroimaging (LONI), University of Southern California
11 BioRep, Milan, Italy
12 University of Pennsylvania, Philadelphia, PA
13 National Institute on Aging, NIH, Bethesda, MD
14 Mount Sinai Beth Israel, New York, NY
15 Indiana University, Indianapolis, IN
16 Emory University of Medicine, Atlanta, GA
17 Oregon Health and Science University, Portland, OR
18 University of Alabama at Birmingham, Birmingham, AL
19 University of South Florida, Tampa, FL
20 Baylor College of Medicine, Houston, TX
21 University of Washington, Seattle, WA
22 Boston University, Boston, MA
23 University of Rochester, Rochester, NY
24 Banner Research Institute, Sun City, AZ
25 Cleveland Clinic, Cleveland, OH
26 University of Tübingen, Tübingen, Germany
27 University of California, San Diego, CA
28 Johns Hopkins University, Baltimore, MD
29 Imperial College of London, London, UK
30 University of Salerno, Salerno, Italy
31 Parkinson's Disease and Movement Disorders Center, Boca Raton, FL
32 University of Cincinnati, Cincinnati, OH
33 Hospital Clinic of Barcelona, Barcelona, Spain
34 Hospital Universitario Donostia, San Sebastian, Spain
35 Tel Aviv Sourasky Medical Center, Tel Aviv, Israel
36 National and Kapodistrian University of Athens, Athens, Greece
37 Columbia University Irving Medical Center, New York, NY
38 Stanford University, Stanford, CA
39 University of Pittsburgh, Pittsburgh, PA
40 Center for Strategy Philanthropy at Milken Institute, Washington D.C.
41 Rutgers University, Robert Wood Johnson Medical School, New Brunswick, New Jersey
42 University of Michigan, Ann Arbor, MI
43 Toronto Western Hospital, Toronto, Canada
44 The Ottawa Hospital, Ottawa, Canada
45 Massachusetts General Hospital, Boston, MA
46 University of Kansas Medical Center, Kansas City, KS
47 University of Southern California, Los Angeles, CA
48 Barrow Neurological Institute, Phoenix, AZ

49 Mayo Clinic Arizona, Scottsdale, AZ
50 University of Colorado, Aurora, CO
51 NYU Langone Medical Center, New York, NY
52 University of Florida, Gainesville, FL
53 Montreal Neurological Institute and Hospital/McGill, Montreal, QC, Canada
54 Cleveland Clinic-Las Vegas Lou Ruvo Center for Brain Health, Las Vegas, NV
55 Clinical Ageing Research Unit, Newcastle, UK
56 John Radcliffe Hospital Oxford and Oxford University, Oxford, UK
57 Universität Lübeck, Luebeck, Germany
58 Radboud University, Nijmegen, Netherlands
59 TransThera Consulting
60 Duke University, Durham, NC
61 Wolfson Institute of Population Health, Queen Mary University of London, UK
62 Philipps-University Marburg, Germany
63 University of Lagos, Nigeria
