# Supplementary figures and images for: Five‐Year Disease Progression in Synuclein Seeding Positive Sporadic Parkinson's Disease
Source: Ann Clin Transl Neurol. 2026 Mar 10:10.1002/acn3.70323. Online ahead of print. doi: 10.1002/acn3.70323 (PMC13393511; doi:10.1002/acn3.70323)

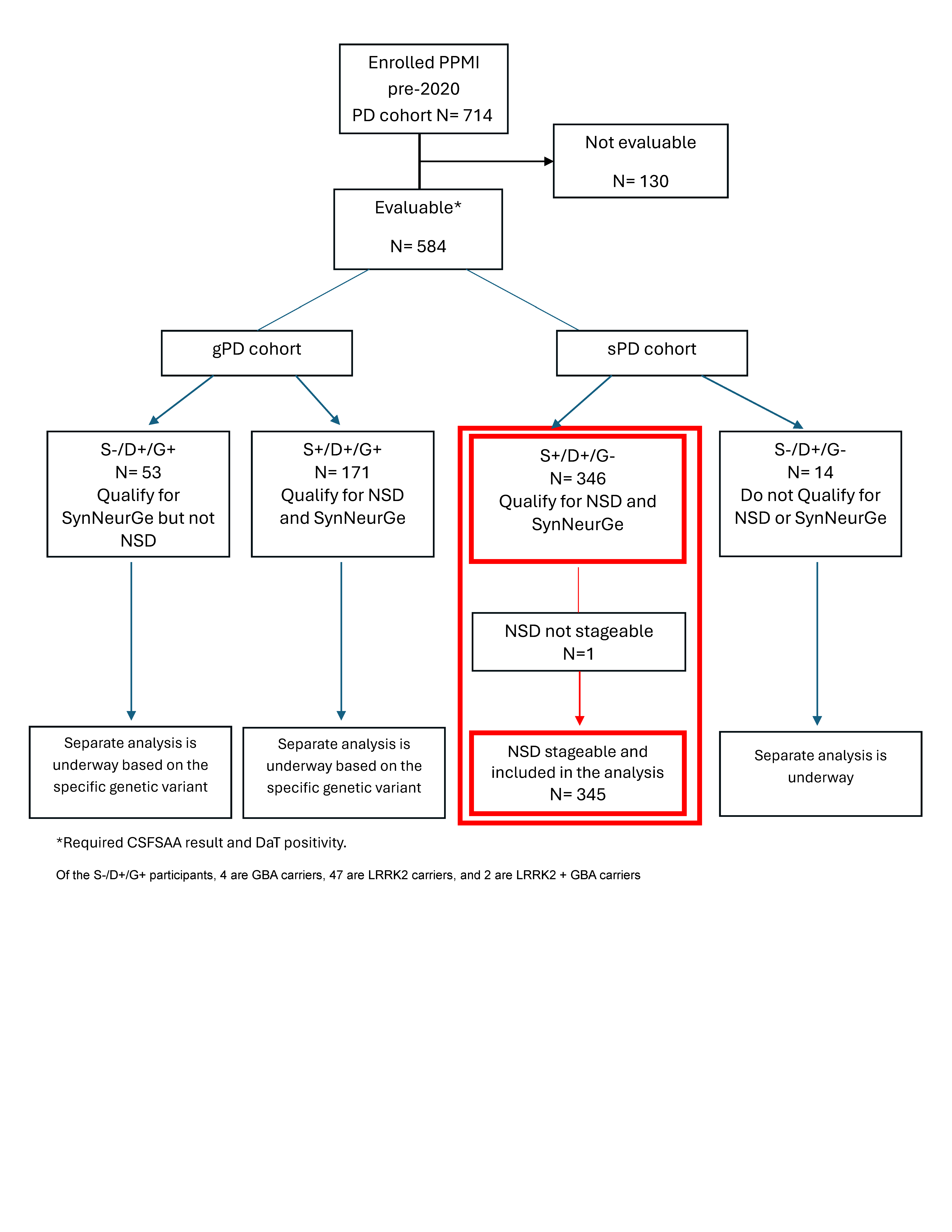

Supplement: Supplementary file 1 — Figure S1: Participant Flowchart Caption: gPD (genetic PD); NSD (Neuronal Synuclein Disease); PD (Parkinson's Disease); sPD (sporadic PD). [file ACN3-9999-0-s010.tif]

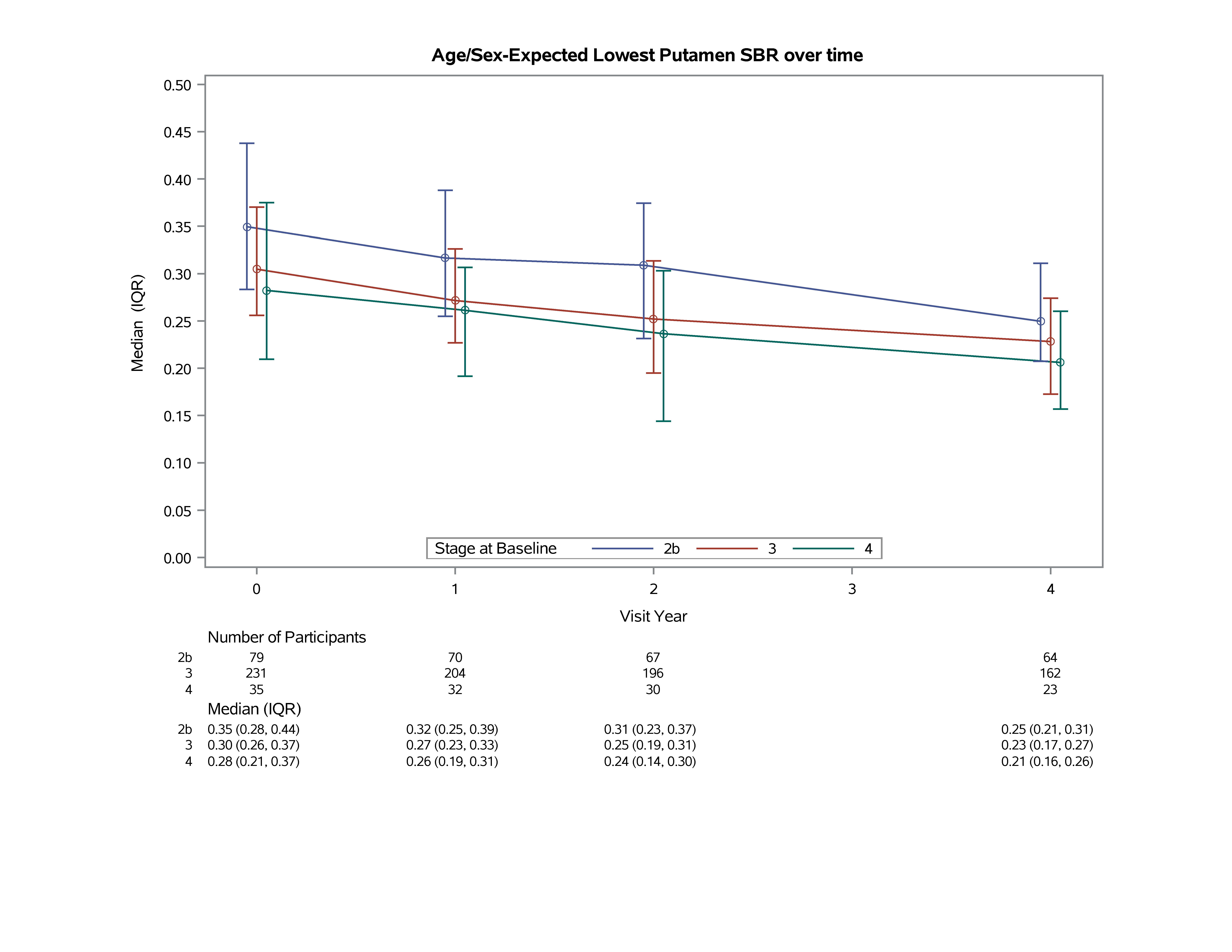

Supplement: Supplementary file 2 — Figure S2a: Age/Sex‐Expected Lowest Putamen SBR over time. [file ACN3-9999-0-s012.tif]

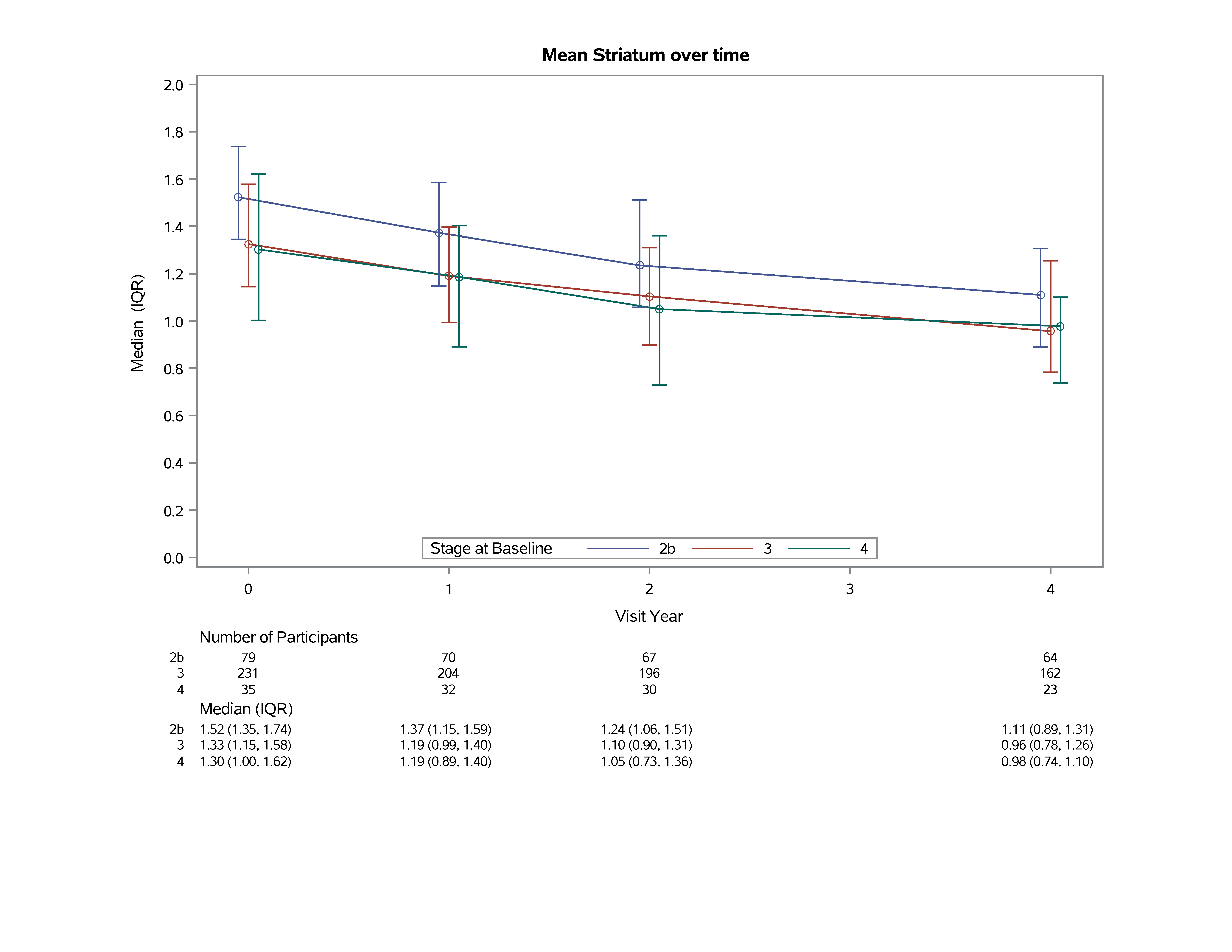

Supplement: Supplementary file 3 — Figure S2b: Mean Striatum Over Time. DAT binding over time. [file ACN3-9999-0-s001.tif]

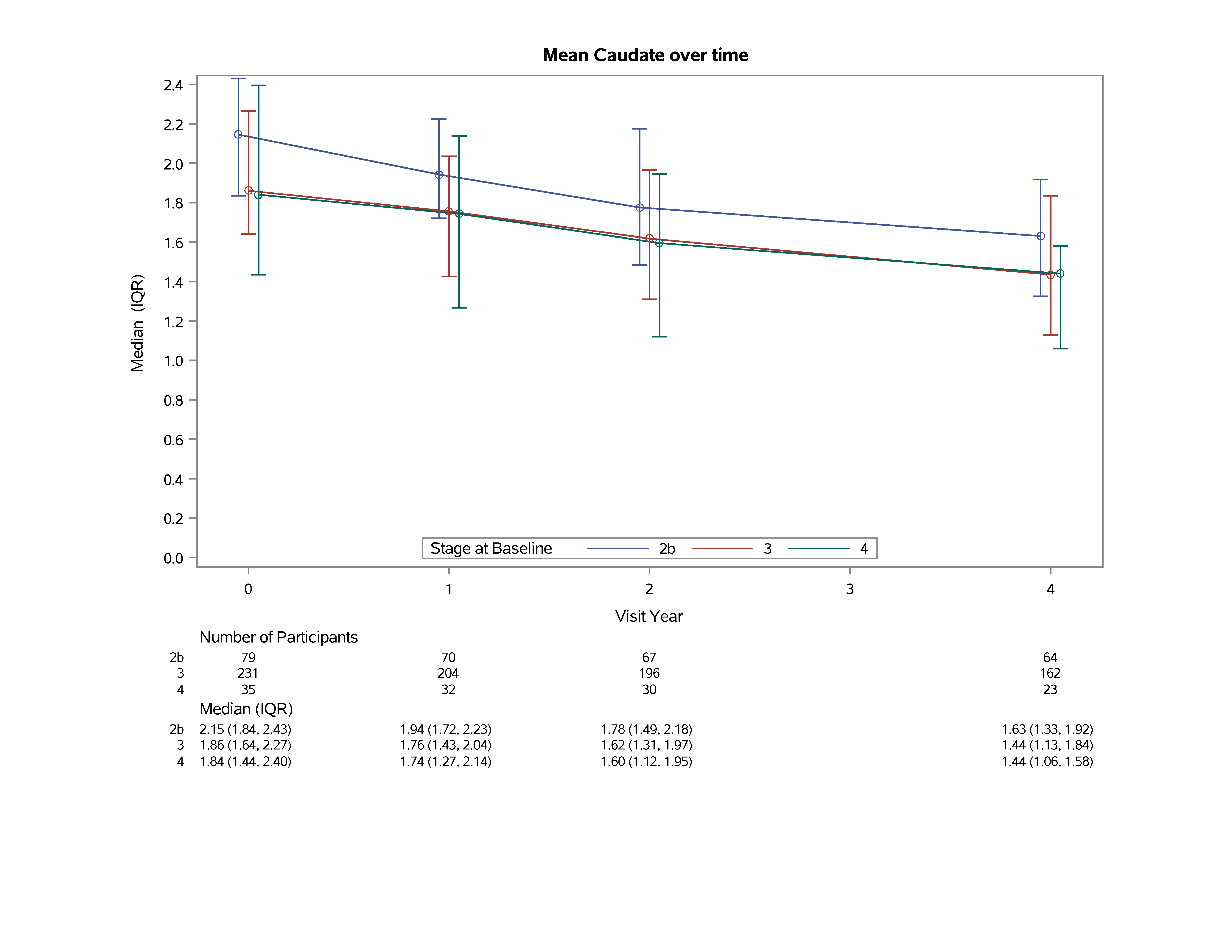

Supplement: Supplementary file 4 — Figure 2c Mean Caudate Over Time. DAT binding over time. [file ACN3-9999-0-s008.tif]

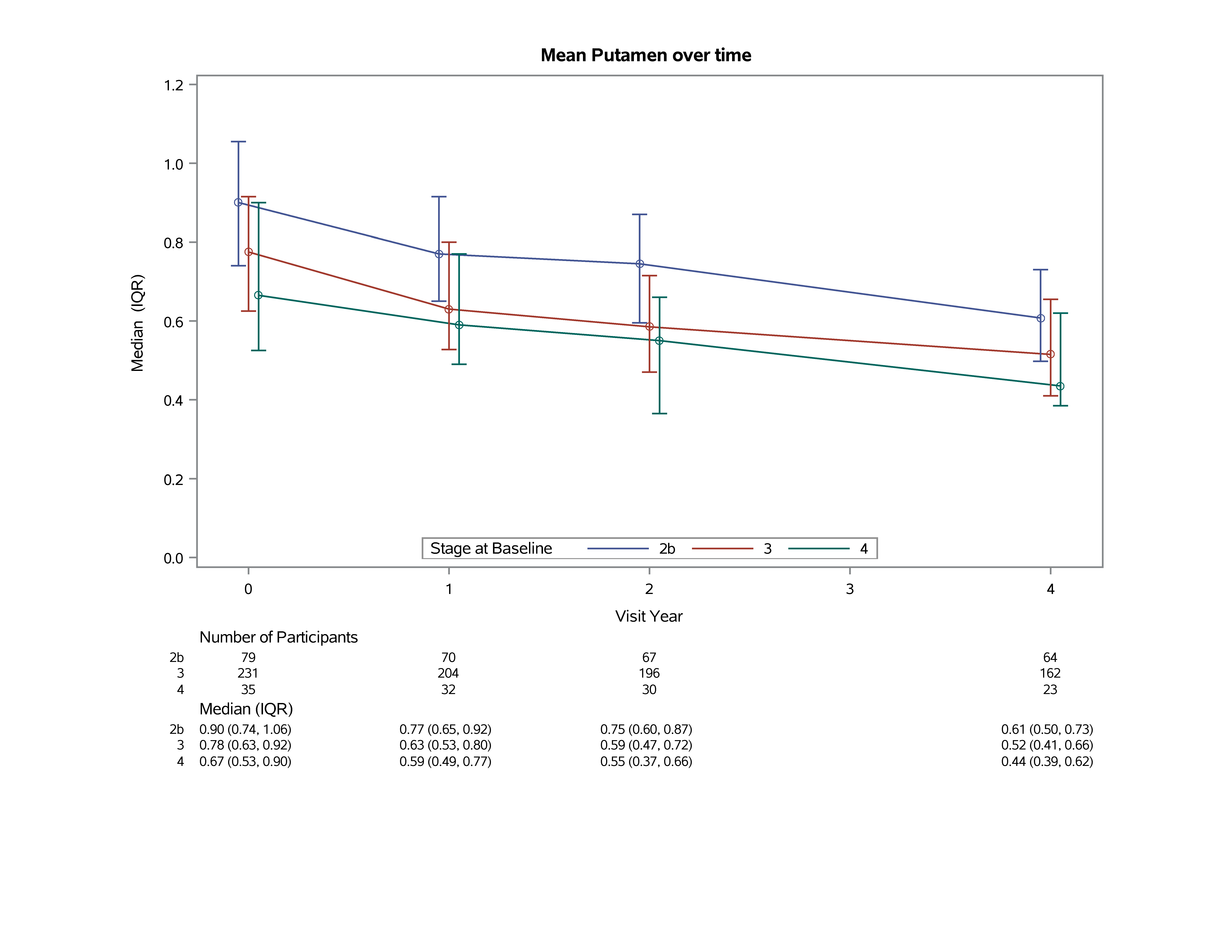

Supplement: Supplementary file 5 — Figure S2d: Mean Putamen Over Time. DAT binding over time. [file ACN3-9999-0-s011.tif]

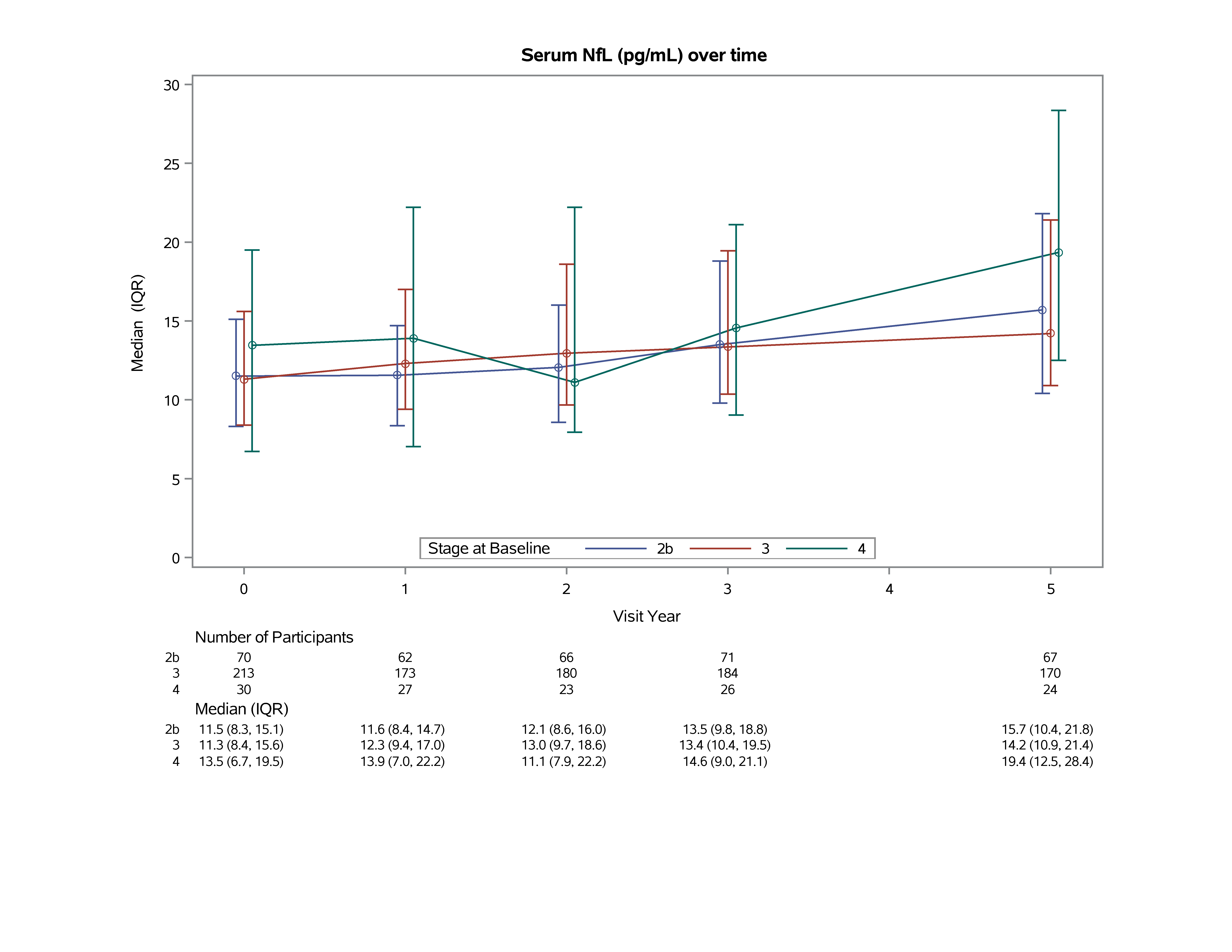

Supplement: Supplementary file 6 — Figure S2e: Serum NfL over time. NfL (Neurofilament ligand). [file ACN3-9999-0-s006.tif]

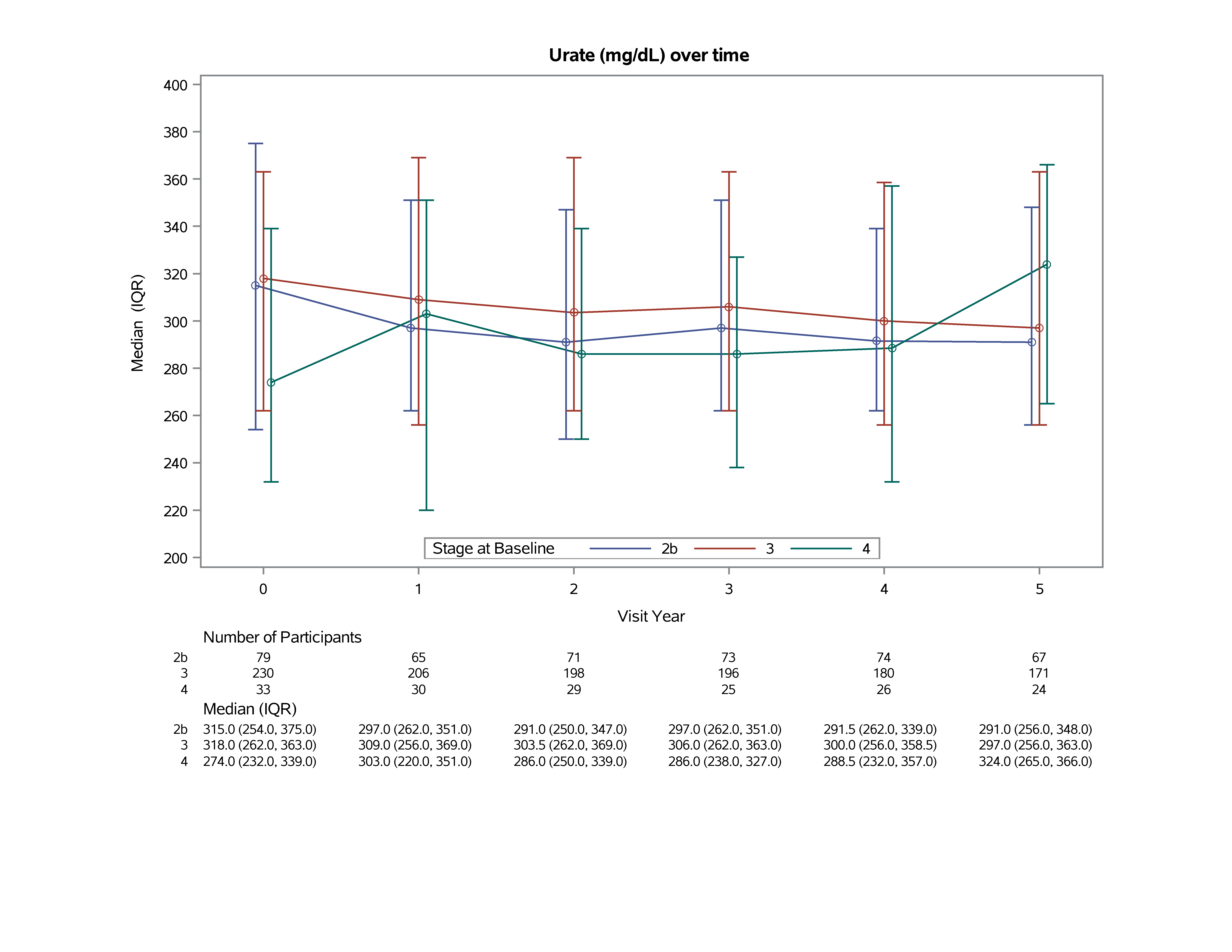

Supplement: Supplementary file 7 — Figure S2f: Urate over time. No caption. [file ACN3-9999-0-s005.tif]

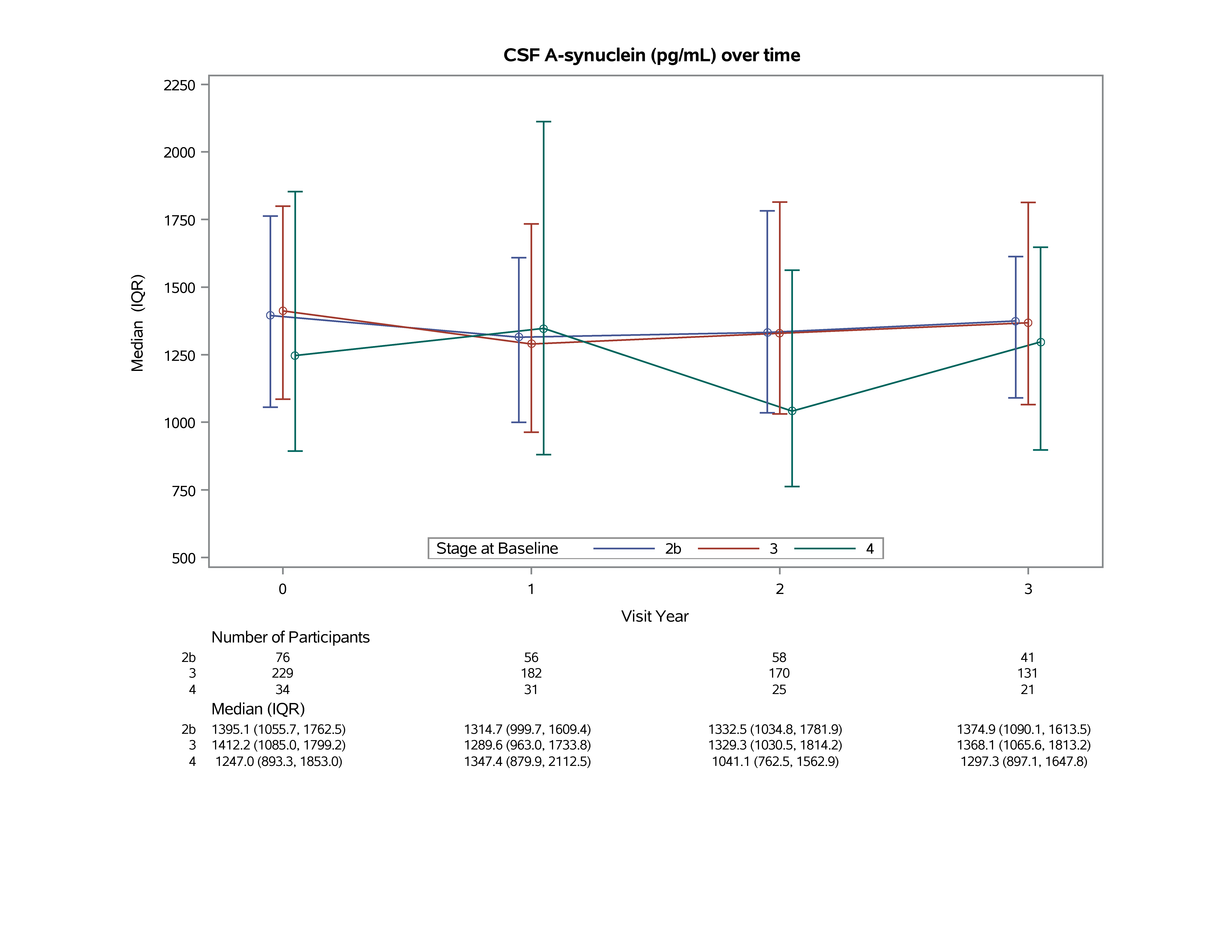

Supplement: Supplementary file 8 — Figure S2g: CSF‐A‐synuclein over time. A‐synuclein (alpha‐synuclein); CSF (cerebrospinal fluid). [file ACN3-9999-0-s009.tif]

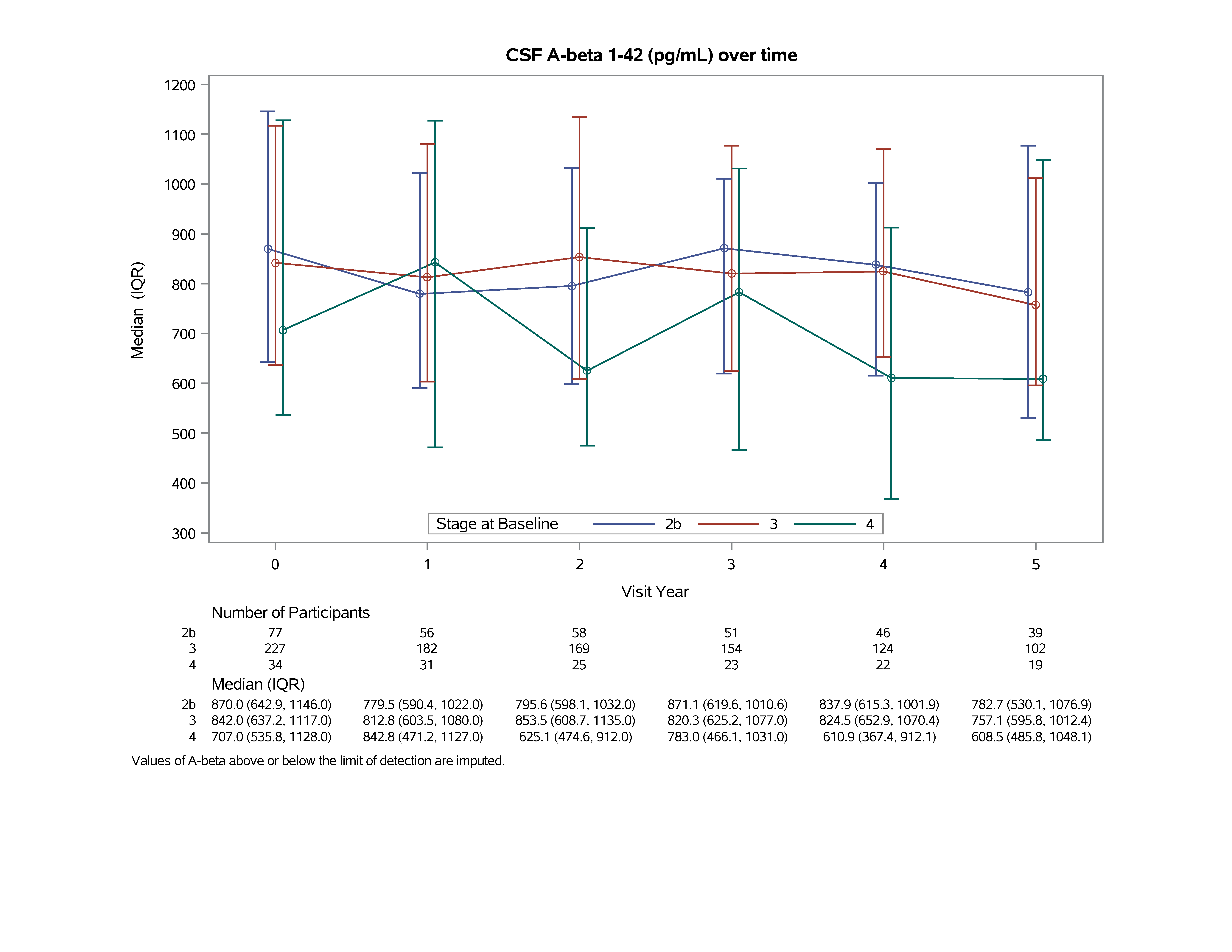

Supplement: Supplementary file 9 — Figure S2h: CSF A‐beta 1–42 over time. A‐beta (amyloid beta); CSF (cerebrospinal fluid). [file ACN3-9999-0-s003.tif]

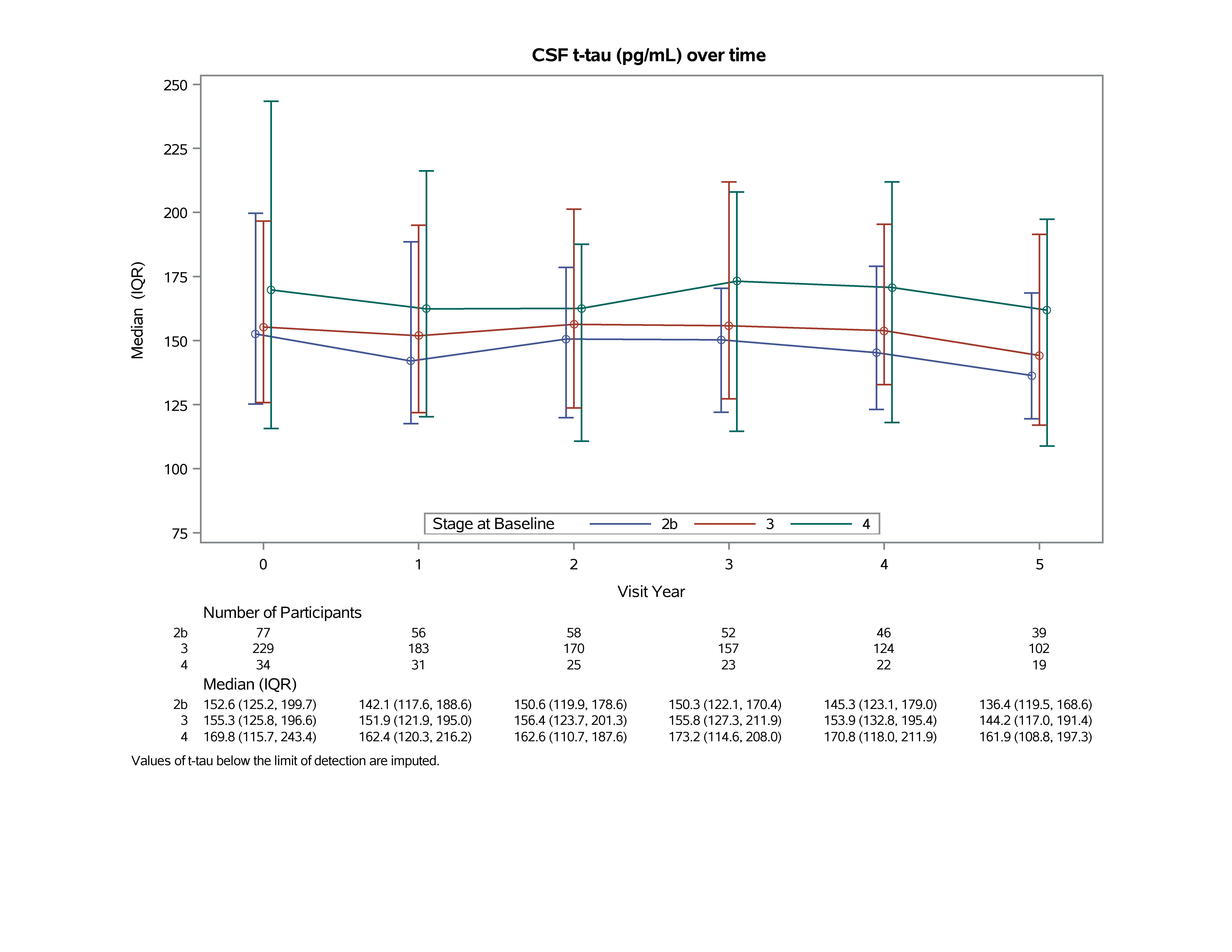

Supplement: Supplementary file 10 — Figure S2i: CSF t‐tau over time. CSF (cerebrospinal fluid); t‐tau (total tau). [file ACN3-9999-0-s004.tif]

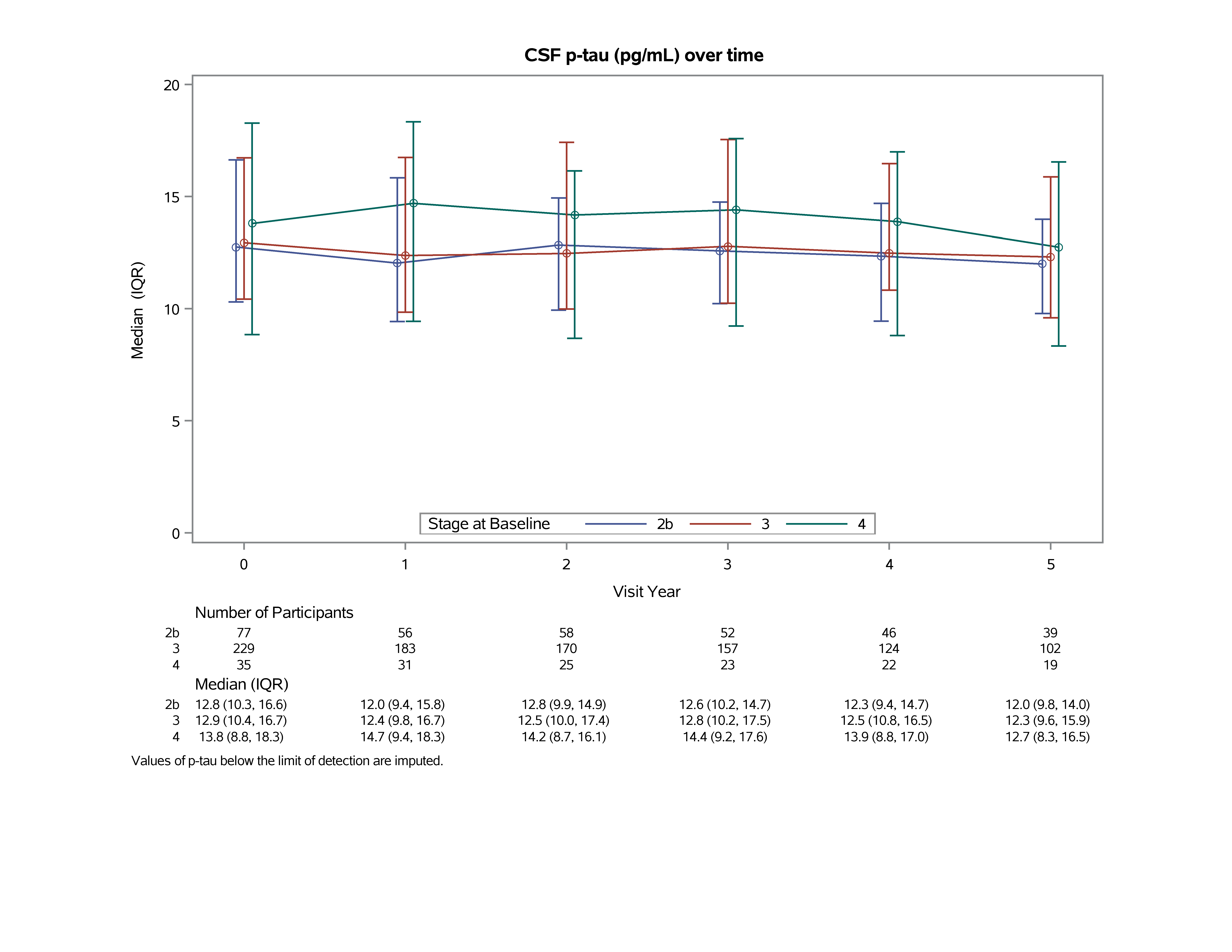

Supplement: Supplementary file 11 — Figure S2j: CSF p‐tau over time. CSF (cerebrospinal fluid); p‐tau (phosphorylated tau). [file ACN3-9999-0-s002.tif]
